# Synthetic analogues of 2-oxo acids discriminate metabolic contribution of the 2-oxoglutarate and 2-oxoadipate dehydrogenases in mammalian cells and tissues

**DOI:** 10.1038/s41598-020-58701-4

**Published:** 2020-02-05

**Authors:** Artem V. Artiukhov, Aneta Grabarska, Ewelina Gumbarewicz, Vasily A. Aleshin, Thilo Kähne, Toshihiro Obata, Alexey V. Kazantsev, Nikolay V. Lukashev, Andrzej Stepulak, Alisdair R. Fernie, Victoria I. Bunik

**Affiliations:** 1 Faculty of Bioengineering and Bioinformatics, Lomonosov Moscow State University, Moscow, Russia; 2 A. N. Belozersky Institute of Physico-Chemical Biology, Lomonosov Moscow State University, Moscow, Russia; 3 Department of Biochemistry and Molecular Biology of Medical University of Lublin, Lublin, Poland; 4 Institute of Experimental Internal Medicine, Otto-von-Guericke University Magdeburg, Magdeburg, Germany; 5 Max Planck Institute of Molecular Plant Physiology, Potsdam, Germany; 6 Faculty of Chemistry, Lomonosov Moscow State University, Moscow, Russia; 7 Present Address: Department of Biochemistry, George W. Beadle Center, University of Nebraska-Lincoln, Lincoln, NE 68588-0664 USA

**Keywords:** Cancer metabolism, Chemical tools, Enzymes, Metabolic pathways, Metabolomics, Small molecules, Biochemistry, Isoenzymes, Metabolomics

## Abstract

The biological significance of the *DHTKD1-*encoded 2-oxoadipate dehydrogenase (OADH) remains obscure due to its catalytic redundancy with the ubiquitous *OGDH*-encoded 2-oxoglutarate dehydrogenase (OGDH). In this work, metabolic contributions of OADH and OGDH are discriminated by exposure of cells/tissues with different *DHTKD1* expression to the synthesized phosphonate analogues of homologous 2-oxodicarboxylates. The saccharopine pathway intermediates and phosphorylated sugars are abundant when cellular expressions of *DHTKD1* and *OGDH* are comparable, while nicotinate and non-phosphorylated sugars are when *DHTKD1* expression is order(s) of magnitude lower than that of *OGDH*. Using succinyl, glutaryl and adipoyl phosphonates on the enzyme preparations from tissues with varied *DHTKD1* expression reveals the contributions of OADH and OGDH to oxidation of 2-oxoadipate and 2-oxoglutarate *in vitro*. In the phosphonates-treated cells with the high and low *DHTKD1* expression, adipate or glutarate, correspondingly, are the most affected metabolites. The marker of fatty acid β-oxidation, adipate, is mostly decreased by the shorter, OGDH-preferring, phosphonate, in agreement with the known OGDH dependence of β-oxidation. The longest, OADH-preferring, phosphonate mostly affects the glutarate level. Coupled decreases in sugars and nicotinate upon the OADH inhibition link the perturbation in glucose homeostasis, known in OADH mutants, to the nicotinate-dependent NAD metabolism.

## Introduction

**Figure 1:**
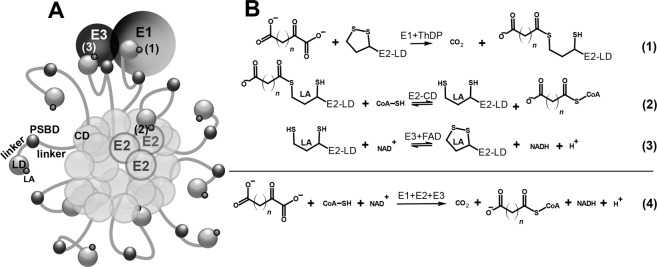
Multienzyme structure and catalysis of 2-oxo acid dehydrogenase complexes allows efficient coupling of several enzymatic reactions. (**A**) Schematic presentation of 2-oxo acid dehydrogenase complexes, comprising a core formed by catalytic domains (CD) of E2 component, and an outer shell of E1 and E3 molecules (shown as single representatives) linked to the core via the peripheral-subunits-binding domains (PSBD) of E2. Highly mobile linkers between CD, PSBD and lipoyl-bearing domain (LD) of E2 allow effective shuttling of LDs with covalently attached lipoamide residues (LA) between the active sites of E1, E2 and E3. (**B**) Enzymatic reactions catalysed by the component enzymes, and overall reactions of OGDHC and OADHC. n = 2 for 2-oxoglutarate, n = 3 for 2-oxoadipate. ThDP – thiamine diphosphate.

2-Oxo acid dehydrogenase complexes comprise a family of multimeric enzymes functioning at the intersections of metabolic pathways involving carbohydrates, lipids and amino acids^1^. The family includes the well-characterized 2-oxoglutarate dehydrogenase complex (OGDHC), which couples the tricarboxylic acid (TCA) cycle with degradation of amino acids of the 2-oxoglutarate group, namely, glutamate, glutamine, arginine, histidine, and proline. The substrate-specific 2-oxoglutarate dehydrogenase (OGDH, EC 1.2.4.1, encoded by the *OGDH* gene, also known as E1o component of the complex), is a well-known and rate-limiting component of OGDHC. The complex also comprises two other types of enzymes: E2o (EC 2.3.1.61) and E3 (EC 1.8.1.4), encoded by the dihydrolipoamide succinyltransferase (*DLST*) and dihydrolipoamide dehydrogenase (*DLD*) genes, respectively. Multiple copies of the E1, E2 and E3 component enzymes form multienzyme complexes schematically exemplified in Fig. 1A. The multimeric structure allows effective coupling of the 2-oxoglutarate oxidative decarboxylation (Fig. 1B, Reaction 1) to the CoA succinylation (Fig. 1B, Reaction 2) and NAD^+^ reduction (Fig. 1B, Reaction 3), resulting in the overall Reaction 4 (Fig. 1B)^1,2^.

In addition to the canonical OGDH, two OGDH isoenzymes with the tissue-specific expression have been characterized, namely the *OGDHL*-encoded 2-oxoglutarate dehydrogenase-like (OGDHL) and *DHTKD1*-encoded dehydrogenase E1 and transketolase domain-containing 1 (DHTKD1) proteins. OGDHL protein is assumed to function as a tissue-specific OGDH isoenzyme^3^, differing from the canonical OGDH by its physiological roles^4–8^. In contrast to the OGDHL isoenzyme of OGDH, the DHTKD1 protein has different substrate specificity, transforming a 2-oxoglutarate homolog with an additional methylene group, 2-oxoadipate, more efficiently than 2-oxoglutarate^3,9,10^. The DHTKD1 role in mammalian 2-oxoadipate oxidation is confirmed by the fact that 2-oxoadipate is accumulated upon human mutations in *DHTKD1* gene^11–13^, despite the ubiquitously expressed OGDHC, which may oxidize 2-oxoadipate at a rate up to 30% of that with 2-oxoglutarate in the purified^14,15^ or reconstituted from recombinant components^9^ states. Thus, the *DHTKD1*-encoded protein is now referred to as 2-oxoadipate dehydrogenase (OADH, E1a), a component of the 2-oxoadipate dehydrogenase complex (OADHC), employing the *DLST*- and *DLD*-encoded proteins as its second- (E2) and third (E3) components^9,16–20^.

Despite the available characteristics of the recombinant complexes^9,21^ and *DHTKD1* mutants in humans and mice^10–13,22–24^, understanding biological significance of *DHTKD1* protein is challenging in view of the known limitations of the *in vitro* studies and highly conditional phenotypes of the *DHTKD1* mutants. Biochemically, the *DHTKD1*-knockout mice show the expected tissue-specific accumulation of 2-oxoadipate and its transamination product, 2-aminoadipate, especially upon the dietary intake of lysine, which is degraded via the OADHC-comprising pathway^24^. Another study of the *DHTKD1* knockout in mice reports both biochemical and physiological manifestations related to the symptoms of Charcot-Marie-Tooth disease type 2Q^23^. However, heterozygous mutations in *DHTKD1* gene are often non-symptomatic and may show neuropathologic or immunopathologic features only under specific conditions, such as presence of disease-associated alleles of other genes or lysine-enriched diet^10,22,25^. The *DHTKD1*-knockout animals do not always exhibit a clinically relevant phenotype either, manifesting general perturbations in homeostasis rather than specific physiological roles of the *DHTKD1*-encoded protein. In particular, the *DHTKD1* expression and/or level of the 2-oxoadipate transamination sibling 2-aminoadipate correlate positively with insulin sensitivity, age and glucose/cholesterol levels in both mice and humans^26–29^. 2-Aminoadipate is also known to upregulate insulin secretion by pancreatic β-cells^26^. Thus, impaired metabolism of 2-oxo- and 2-aminoadipate upon mutations of the DHTKD1 protein in animals may dysregulate glucose metabolism, decreasing physiological fitness of an organism. Besides, 2-aminoadipate is known to be toxic for astrocytes^30,31^, although in models of Parkinson’s disease it is protective^32,33^. These controversial actions of 2-aminoadipate may be due to structural similarity between 2-aminoadipate and glutamate, which may enable 2-aminoadipate to decrease the excitotoxic glutamate release under pathological conditions, but interfere with normal regulation of glutamate neurotransmission^30,31,34^. In good accord with such a complexity, the phenotypes and adaptations of human and animal organisms to the *DHTKD1* mutations *in vivo* are highly conditional, obscuring specific molecular mechanisms linking the OADH-catalysed reaction to the decreased organismal fitness. As a result, no metabolic alterations other than the immediate and expected consequences of the downregulated OADH reaction are described. Both *DHTKD1* knockout animals and mutant patients may show accumulation of 2-oxoadipate, 2-aminoadipate and 2-hydroxyadipate in urine and/or plasma^11–13,23^. The findings indicate that the impaired oxidation of 2-oxoadipate by OADH is not compensated by ubiquitously expressed OGDH, despite a rather high level of the OGDH activity in reaction with 2-oxoadipate *in vitro*. Delineation of the physiological role(s) of the *DHTKD1*-encoded protein in *in vivo* studies is complicated by tissue specificity of the *DHTKD1*-dependent changes^24^, significant variation in the *DHTKD1* expression in a population^27,35^ and specific environmental conditions when the effects of the *DHTKD1* expression acquire significance^24,27^. For instance, in liver, the *DHTKD1*-encoded protein is shown to be a primary regulator of 2-aminoadipate^27^, but this may be different in other tissues. In fact, the *DHTKD1* knockout in mice elevated 2-oxoadipate and 2-aminoadipate in liver, but did not affect these metabolites in the brain^24^. As a result, understanding the organization and significance of the OADHC-involving pathway(s) obviously requires studies at a less complicated than organismal level. Therefore, in this work the problem is addressed using metabolomics of cultured cells with natural variations of the *DHTKD1* expression, supported by *in vitro* studies of the enzymes from the rat tissues with similar variations in the *DHTKD1* expression.

To further develop tools for discriminating biological functions of the *OGDH(L)* and *DHTKD1* gene products, we have taken into account the successful application of the synthetic OGDH inhibitor succinyl phosphonate (SP) for the enzyme regulation *in vivo*^1,36,37^ and the better accommodation of bulkier substrates by the active site of OADH, compared to OGDH^3,38^. For the present work, a series of SP homologues have been synthesized to compare their inhibitory potential towards OGDHC and OADHC *in vitro* and in cell cultures. Activities of the enzyme-enriched fractions along with metabolism and viability of the control and phosphonates-treated cultured cells have been assessed in the systems with varied OADH expression levels. Comparative analysis of the action of the homologous phosphonates indicates that the longest of the substrate analogues, adipoyl phosphonate (AP), is a specific *in vivo* inhibitor of the *DHTKD1*-encoded protein. Metabolomics experiments reveal that cells with varied *DHTKD1* expression possess specific metabolic features and respond differently to the phosphonate analogues of 2-oxoglutarate and 2-oxoadipate. Based on the metabolomics data of the control and phosphonates-treated cells, specific cellular action of the OADH inhibitor AP is shown, in good accord with the inhibition studies *in vitro*. Unlike SP and GP, AP is able to decrease cellular glutarate and glucose. Based on the coupled decrease in cellular glutarate, glucose and nicotinic acid after inhibition of OADH by AP, the nicotinate-dependent NAD metabolism is suggested to mediate the perturbed levels of glucose. Thus, the synthesis and specific action of the OADH inhibitor AP, characterized in this work, provide a useful tool for deciphering cell-specific molecular mechanisms which underlie the known associations between the *DHTKD1* expression with diabetes, obesity and cancer.

## Results

### Activity and stability of OADHC in tissue homogenates depends on relative expression of enzymatic components of the OGDH and OADH complexes

**Figure 2:**
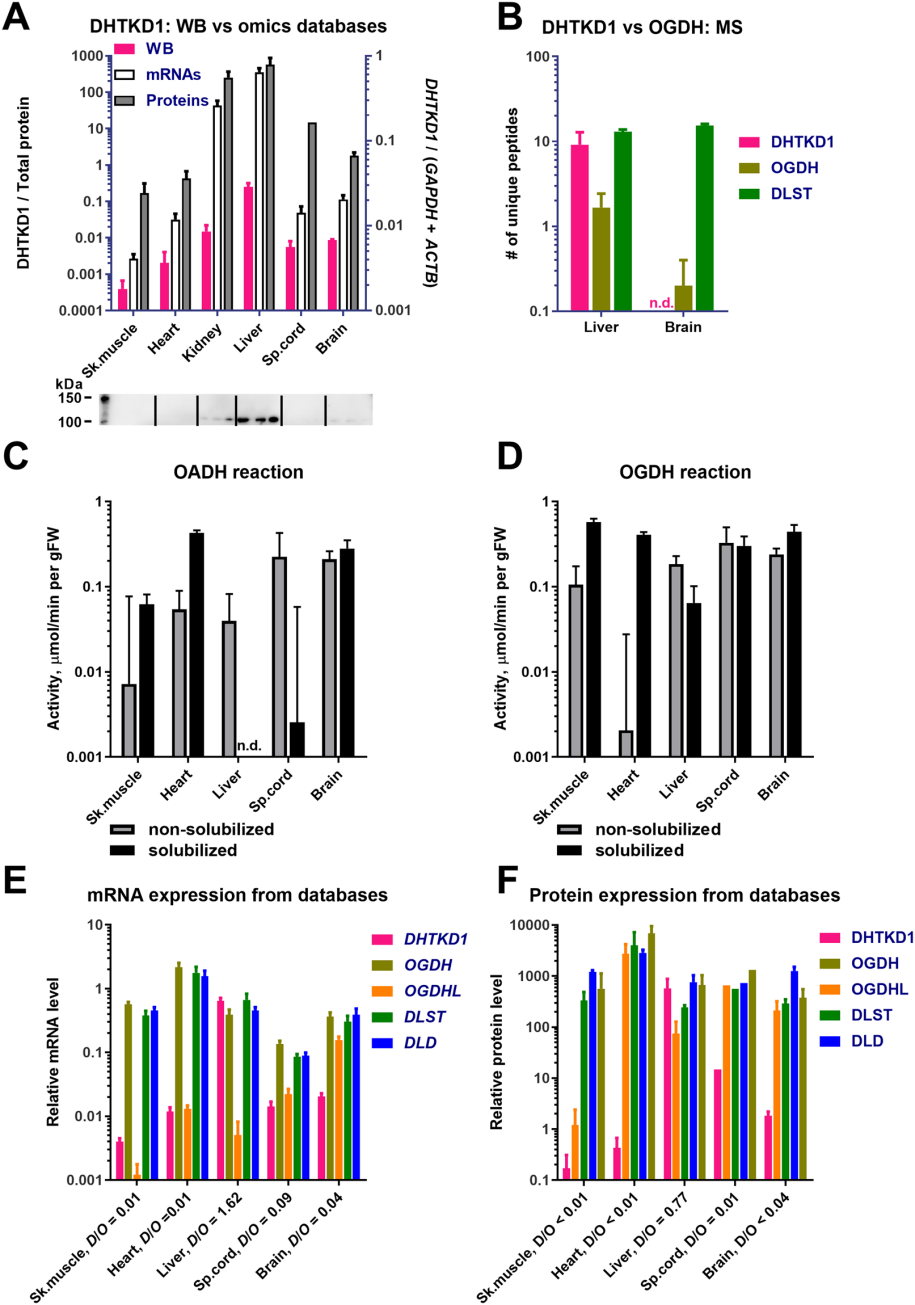
Levels of the proteins, mRNA and activities of OADHC and OGDHC in different rat tissues. (**A**) Comparison of *DHTKD1* expression, determined by Western blot (WB) in our experiments, and extracted from transcriptomics and proteomics databases. The tissue homogenates (n = 3 for each tissue) reacted with anti-DHTKD1 antibodies (Invitrogen). Pink bars correspond to the intensities of bands in 100–110 kDa area (Fig. S1A), which were normalized to the total protein in the lane, measured by 2,2,2-trichloroethanol staining (Fig. S1B). The data extraction from the gene expression databases is described in Materials and Methods. (**B**) Mass-spectrometric detection of the DHTKD1, OGDH, and DLST proteins in mitochondria isolated from the rat liver (n = 6) or cerebral cortex (n = 5). Numbers of unique peptides, detected after in-gel trypsin digestion, are presented. (C,D) Specific activities of the rat tissue homogenates in catalysing the OADH (**C**) and OGDH reactions (**D**), assayed before (grey bars) or after solubilization of membranous compartments by sonication and detergents (black bars). (**E**) Relative levels of mRNAs for the multienzyme complex components *DHTKD1*, *OGDH*, *OGDHL*, *DLST* and *DLD* in the rat tissues. The transcriptomic data are extracted from ≥18 independent series of experiments for each tissue. (**F**) Relative levels of the DHTKD1, OGDH, OGDHL, DLST and DLD proteins in the mouse tissues. Except for spinal cord (1 experiment), the protein abundances were extracted from ≥2 independent experiments. D/O is the ratio between the expression of *DHTKD1* and *OGDH* + *OGDHL* genes at the mRNA and protein levels. n.d. – Not determined.

To choose the best source for assays of the *DHTKD1*-encoded protein and differentiation of its activity from that of OGDH(L), the relative abundance of the enzymes in different tissues has been analysed. The western blot detection of the *DHTKD1* protein (OADH) agrees well with the transcriptomics and proteomics data (Fig. 2A), and our own mass-spectrometry estimations (Fig. 2B). All the approaches demonstrate that the *DHTKD1* protein is expressed in the liver much more than in the other tissues shown in Fig. 2. In particular, the OADH-specific peptides are much better detectable in homogenates of the rat liver than brain, whereas the OGDH- and DLST-specific peptides are equally detectable in both tissues (Fig. 2B).

Catalysis of the overall OADH (Fig. 2C) or OGDH (Fig. 2D) reactions in different tissues requires the assembly of the multienzyme complexes formed by non-covalent interactions of multiple copies of three catalytic subunits (Fig. 1). It is therefore not surprising that the assayed activities (Fig. 2C,D) do not correspond to the expression of only the OADH and OGDH components of the complexes (Fig. 2E,F). Obviously, the activities depend on the expression of all the components of the complexes (Fig. 2E,F). It is known that the expression of components of multienzyme structures *in vivo* occurs at the ratios enabling successful formation of the needed levels of the multienzyme structures^39^. Competition of the OADH and OGDH components for their common partner, the *DLST*-encoded E2o component, also implies that the components ratios should be in accord with relative affinities of OADH and OGDH to E2o, which are known to differ^38,40^. Transcriptomics (Fig. 2E) and proteomics (Fig. 2F) data indicate that in all the tissues the expression of E2o and E3 is comparable to the expression of the *OGDH* gene, and does not significantly increase when the *DHTKD1* expression approaches that of *OGDH*. The ensuing competition between the OGDH and OADH components of the multienzyme complexes for the same *DLST*-encoded E2o is obvious from our experimental data. At very similar expressions of the *OGDH*, *DLST*, and *DLD* transcripts (Fig. 2E) and proteins (Fig. 2F), the fully solubilized OGDHC activity in liver is an order of magnitude lower than that in skeletal muscles (black bars in Fig. 2D). This is obviously due to the stronger competition between the *OGDH* and *DHTKD1*-encoded proteins for E2o in the liver, where the expression of the two proteins is comparable, than in the skeletal muscles, where *OGDH* is expressed much more than *DHTKD1* (Fig. 2E,F).

**Figure 3:**
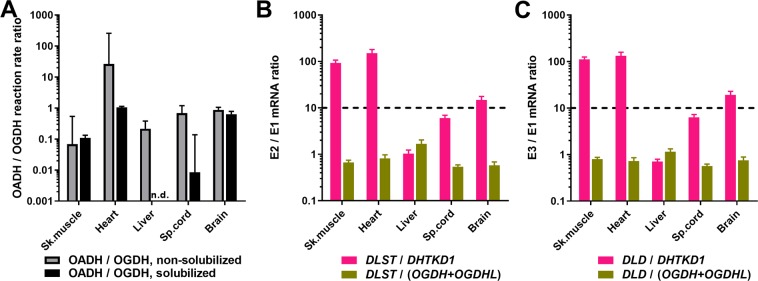
Relative abundances of the OGDHC and OADHC components in different rat tissues correspond to different stabilities of the complexes upon solubilization of mitochondrial proteins by sonication and detergents. (**A**) Ratios of the activities of the rat tissue homogenates in catalysing the OADH and OGDH reactions before (grey bars) and after (black bars) the solubilization procedure. (**B**) Ratios of mRNA levels for the second and the first enzymatic components of OADHC and OGDHC. (**C**) Ratios of mRNA levels for the third and the first enzymatic components of OADHC and OGDHC. n.d. – Not determined.

The competition between the OADH and OGDH components for binding site on E2o may also lead to a lower stability of the overall OADHC *vs* OGDHC activity under conditions promoting dissociation of the complexes. This may occur upon solubilization of mitochondrial proteins by sonication and detergents. Comparison of the tissue expression of the components of the complexes (Fig. 2E,F) with their relative stabilities to the dissociating factors (Fig. 2C,D), reveals the components ratios under which the OADHC activity is better preserved (Figs. 2C and 3A). This is observed when the *DLST*/*DHTKD1* and *DLD*/*DHTKD1* ratios (i) are higher than 10 and (ii) exceed at least by an order of magnitude the *DLST*/*OGDH(L)* and *DLD*/*OGDH(L)* ratios (Fig. 3B,C). In other words, at a comparable expression of the OGDH and E2o components of the multienzyme complexes, which is observed in all the tissues (Fig. 2E,F), at least a 10-fold excess of E2o over OADH is required to preserve the OADHC activity during the mitochondrial solubilization. In contrast, the activity of OGDHC is not lost during the solubilization at the comparable expression of OGDHC and E2o, given the expression of *DHTKD1* is low (Fig. 2D). Pointing to a much weaker binding of E2o to OADH (E1a) than to OGDH (E1o), these data are in good accordance with the differences of the E2-binding regions between the *DHTKD1-* and *OGDH*-encoded protein sequences^3^ and the relative binding affinities recently shown in studies of the recombinant proteins^38,40^.

According to the data on the recombinant OADHC and OGDHC^9,21^, the 2-oxo substrate concentrations (2 mM) in our assays of homogenates are saturating. Hence, the assayed activities of OADHC and OGDHC (Fig. 2C,D) correspond to the maximal rates of the overall OADH and OGDH reactions, catalysed by the complexes in the analysed samples. Our data thus reveal that OADHC and OGDHC in mammalian tissues possess a much stronger specificity to their cognate 2-oxo substrates, compared to the 20–30% cross-reactivity of the recombinant complexes, based on the *k*_*cat*_ values with these substrates^9,21^. In fact, in the heart tissue before the solubilization by sonication and detergents, the OADH reaction is well-measurable, whereas the OGDH reaction rate is only about 3% of the OADH reaction rate (Fig. 2C,D). In contrast, when the proteins are fully solubilized by sonication and detergents, the preparation may possess a high OGDHC activity devoid of the OADHC activity, as in the liver and spinal cord (Fig. 2C,D). The different substrate specificity of the native and recombinant complexes may be due to the artificial structures of the recombinant complexes, which have been obtained at the ratios of the components, strongly deviating from those known for the native OGDHC^9,21^.

### Analysis of the inhibition of OADH and OGDH reactions by the phosphonate analogues of homologous 2-oxodicarboxylic acids

**Figure 4:**
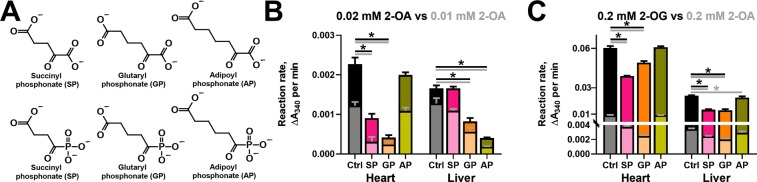
*In vitro* inhibition of OGDHC and OADHC from the rat heart and liver by 0.01 mM phosphonate analogues of 2-oxodicarboxylates at physiologically relevant concentrations of 2-oxo substrates. (**A**) The 2-oxo acid substrates and their phosphonate analogues. (**B**) Inhibition of the rates of the OADH reaction at 0.01 or 0.02 mM 2-oxoadipate (2-OA) by 0.01 mM succinyl, glutaryl or adipoyl phosphonates (SP, GP or AP, correspondingly). (**C**) Inhibition of the rates of the OGDH or OADH reactions at 0.2 mM 2-oxoglutarate (2-OG) or 2-oxoadipate (2-OA) by 0.01 mM SP, GP or AP. The reactions were started by the enzyme addition to the reaction medium. App. 40 µg or 65 µg of protein of the heart or liver preparations, correspondingly, was added per well. The control (Ctrl) reaction rates were measured in the media omitting the inhibitors. The lower reaction rates (with 0.01 (**B**) or 0.2 mM (**C**) 2-OA) and corresponding significant differences are depicted with lighter shades of the same colours. Statistical significances were analysed by one-way ANOVA for multiple groups comparison, followed by Tukey’s post-hoc test. For the simplicity of the presentation, only the significant differences (p ≤ 0.05) *vs* the control reaction rates are shown by asterisks.

For the kinetic analysis of the inhibition of the OADH and OGDH reactions by the phosphonate analogues of their 2-oxo acid substrates (Fig. 4A), rat tissues with different OADH expression have been fractionated to enrich the studied activities. To preserve the OADHC activity, the solubilization of mitochondrial complexes was done by 3% Triton X-100 without sonication. The inhibitory potential of the phosphonates towards OADHC and OGDHC has been compared at the physiologically relevant levels of 2-oxo substrates (Fig. 4). In mammalian tissues, the concentrations of 2-oxoglutarate are about 0.1–0.2 mM^41^, while 2-oxoadipate normally does not exceed 0.01 mM^11,24^. Due to low reaction rates at this concentration of 2-oxoadipate, we determined the inhibition also at 0.02 mM substrate (Fig. 4B). We also compared the relative inhibitory power of the phosphonates at a fixed (0.2 mM) concentration of 2-oxoglutarate *vs* 2-oxoadipate (Fig. 4C).

The results in Fig. 4 demonstrate that the relative inhibitory power of the phosphonates in the OADH and OGDH reactions strongly depends on the relative expression of the *DHTKD1* protein, which is up to three orders of magnitude higher in the rat liver, compared to the heart (Fig. 2A). In fact, at 0.01–0.02 mM 2-oxoadipate, the longest of the phosphonates, AP, does not inhibit the OADH reaction catalysed by the cardiac preparation with a low *DHTKD1* expression, but does affect this reaction in case of catalysis by the liver preparation with a high *DHTKD1* expression (Fig. 4B). Under the same conditions, the shortest of the phosphonates, SP, exhibits the opposite behaviour, significantly inhibiting only the OADH reaction rate catalysed by the cardiac preparation (Fig. 4B). The intermediate length of GP causes its reactivity in the OADH reaction catalysed by both the cardiac and liver preparations (Fig. 4B). These data show that AP and SP discriminate the active sites of OGDH and OADH better, than GP. Besides, one may conclude that at the low concentrations of 2-oxoadipate the OADH reaction is predominantly catalysed by different enzyme complexes, i.e. OADHC and OGDHC, in the liver and heart preparations, correspondingly.

Unlike the tissue-specific action of the phosphonates on the OADH reaction (Fig. 4B), the pattern of inhibition of the OGDH reaction does not strongly depend on the tissue, with SP and GP significantly inhibiting the OGDH reaction catalysed by both the cardiac and hepatic preparations (Fig. 4C). Remarkably, with both tissues, the inhibitory action of AP is absent in the OGDH reaction at 0.2 mM 2-oxoglutarate (Fig. 4C). However, AP still inhibits the OADH reaction at 0.2 mM 2-oxoadipate, if this is catalysed by the liver preparation (Fig. 4C). A higher contribution of OADHC in the liver, compared to heart, preparation to the catalysis of OADH reaction at 0.2 mM 2-oxoadipate is also obvious by the degree of the inhibition of this reaction by SP and GP. At 0.2 mM 2-oxoadipate, the OADH reaction catalysed by cardiac preparation is significantly inhibited by SP (60%) and GP (75%) much more than the same reaction catalysed by the liver preparation, where the inhibition by SP and GP is only 30% (Fig. 4C).

Remarkably, as a competitive inhibitor of OGDHC, SP should be more inhibitory at lower 2-oxoadipate concentrations. This is true for the cardiac preparation, but with the hepatic one the inhibitory power of SP increases along with the elevation of the 2-oxoadipate concentration from 0.02 mM (no observed inhibition by SP) to 0.2 mM (30% inhibition by SP) (Fig. 4B,C). The finding indicates a higher contribution of the SP-inhibited OGDHC to the OADH reaction at the non-physiological 2-oxoadipate concentration (0.2 mM), in good accord with a lower affinity of OGDHC *vs* OADHC to 2-oxoadipate^9,21^.

Thus, the inhibitory action of AP and SP demonstrates their preferential binding to OADH (*DHTKD1*-encoded protein) and OGDH (*OGDH*(*L*)-encoded protein), correspondingly, whereas efficiency of the OGDH and OADH inhibition by GP depends on the substrate, its concentration and relative expression of OGDH and OADH.

### Study of the interactions of the homologous phosphonates with other enzymes transforming 2-oxo acids or their analogues

When SP was introduced as a highly specific OGDHC inhibitor *in vivo*, its reactivity to other enzymes transforming 2-oxoglutarate and its structural analogues was studied to ensure the specific action on OGDHC^42,43^. To confirm the specificity of the SP homologues introduced in this work, we have performed similar studies with GP and AP. Comparison of the influence of the three phosphonates on the activities of different enzymes of central metabolism transforming 2-oxo acids and/or their analogues, is presented in Table 1. The concentration of the phosphonates used in these studies (0.1 mM) causes strong inhibition of OGDHC by SP and OADHC by AP at the physiological levels of the substrates (Table 1). The comparable concentration of the 2-oxo/2-hydroxy substrates (0.2 mM) was used to study potential off-target effects, unless the reliable activity assay required a higher concentration. For instance, 2 mM 2-oxoglutarate was required to assay the transaminase reactions, whose K_m_ for 2-oxoglutarate is in the mM range^44–46^. The results of Table 1 confirm the previously known specificity of SP^37,42,43^ and indicate that the activities of the enzymes from different classes are not significantly affected by the new phosphonates AP and GP either.

**Table 1:** Effects of 0.1 mM SP, GP and AP on the activities of the enzymes of central metabolism, transforming 2-oxo acids and/or their analogues.

Enzyme	Substrate concentration	Source	Specific activities			
			Ctrl	0.1 mM SP	0.1 mM GP	0.1 mM AP
OADHC/OGDHC	0.01 mM 2-oxoadipate	Enzyme-enriched fraction from rat liver	0.96 ± 0.10	0.59 ± 0.04	0	0.09 ± 0.01
	0.2 mM 2-oxoglutarate		14.9 ± 0.4	2.5 ± 0.2	0.2 ± 0.2	12.1 ± 0.5
	0.01 mM 2-oxoadipate	Enzyme-enriched fraction from rat heart	1.23 ± 0.04	0	0	0.87 ± 0.36
	0.2 mM 2-oxoglutarate		123 ± 1	50 ± 3	52 ± 1	119 ± 2
PDHC	0.2 mM pyruvate	Enzyme-enriched fraction from rat heart	163 ± 4	148 ± 2	144 ± 1	156 ± 7
BCDHC	0.2 mM 3-methyl-2-oxovalerate	Enzyme-enriched fraction from rat liver	1.81 ± 0.05	1.70 ± 0.06	1.75 ± 0.04	1.93 ± 0.11
GDH	0.2 mM 2-oxoglutarate	Purified from bovine liver	81.9 ± 1.3	74.8 ± 0.5	69.3 ± 0.8	73.7 ± 3.1
		Rat liver homogenate	27.3 ± 0.4	28.4 ± 0.3	28.3 ± 0.0	26.3 ± 0.4
MDH	0.2 mM oxaloacetate	Rat liver homogenate	345 ± 11	315 ± 22	369 ± 14	379 ± 11
		Rat heart homogenate	471 ± 5	500 ± 8	518 ± 7	517 ± 7
LDH	0.2 mM lactate	Purified from rabbit muscle	219 ± 1	217 ± 2	215 ± 8	211 ± 5
PK	0.2 mM phospho-enolpyruvate	Purified from rabbit muscle	27.3 ± 1.0	24.1 ± 0.3	25.6 ± 0.8	26.9 ± 1.4
GOT	2 mM 2-oxoglutarate	Rat liver homogenate	16.7 ± 0.7	19.6 ± 0.2	20.3 ± 0.2	21.4 ± 0.4
		Rat heart homogenate	30.4 ± 0.6	31.7 ± 1.2	28.7 ± 1.0	30.9 ± 0.3
GPT		Rat liver homogenate	1.83 ± 0.06	1.74 ± 0.41	1.49 ± 0.23	1.91 ± 0.10
NADP^+^-IDH	2 mM isocitrate	Rat liver homogenate	5.30 ± 0.11	4.31 ± 0.06	5.13 ± 0.03	5.36 ± 0.03
		Rat heart homogenate	1.20 ± 0.02	1.00 ± 0.01	1.29 ± 0.11	1.28 ± 0.04
NADP^+^-ME	2 mM malate	Rat liver homogenate	0.085 ± 0.002	0.067 ± 0.002	0.078 ± 0.005	0.102 ± 0.002

All enzyme assays were performed under standard assay conditions (see Material and Methods) at the indicated concentrations of the 2-oxo substrates or their analogues. Specific activities of the enzymes are given as mean ± SEM in nmol/min per mg of protein for the enzyme-enriched fractions, in µmol/min per mg of protein for purified enzymes, or in µmol/min per g of tissue fresh weight (gFW) for homogenates. The following abbreviations are used: OADHC – 2-oxoadipate dehydrogenase complex, OGDHC – 2-oxoglutarate dehydrogenase complex, PDHC – pyruvate dehydrogenase complex, BCDHC – branched-chain 2-oxo acid dehydrogenase complex, GDH – glutamate dehydrogenase, MDH – malate dehydrogenase, LDH – lactate dehydrogenase, PK – pyruvate kinase, GOT – glutamate oxaloacetate transaminase, GPT – glutamate pyruvate transaminase, NADP^+^-IDH – NADP^+^-dependent isocitrate dehydrogenase, NADP^+^-ME – NADP^+^-dependent malic enzyme.

### Different *DHTKD1* expression in cell lines correlates with different abundance of the saccharopine pathway intermediates or nicotinate

**Figure 5:**
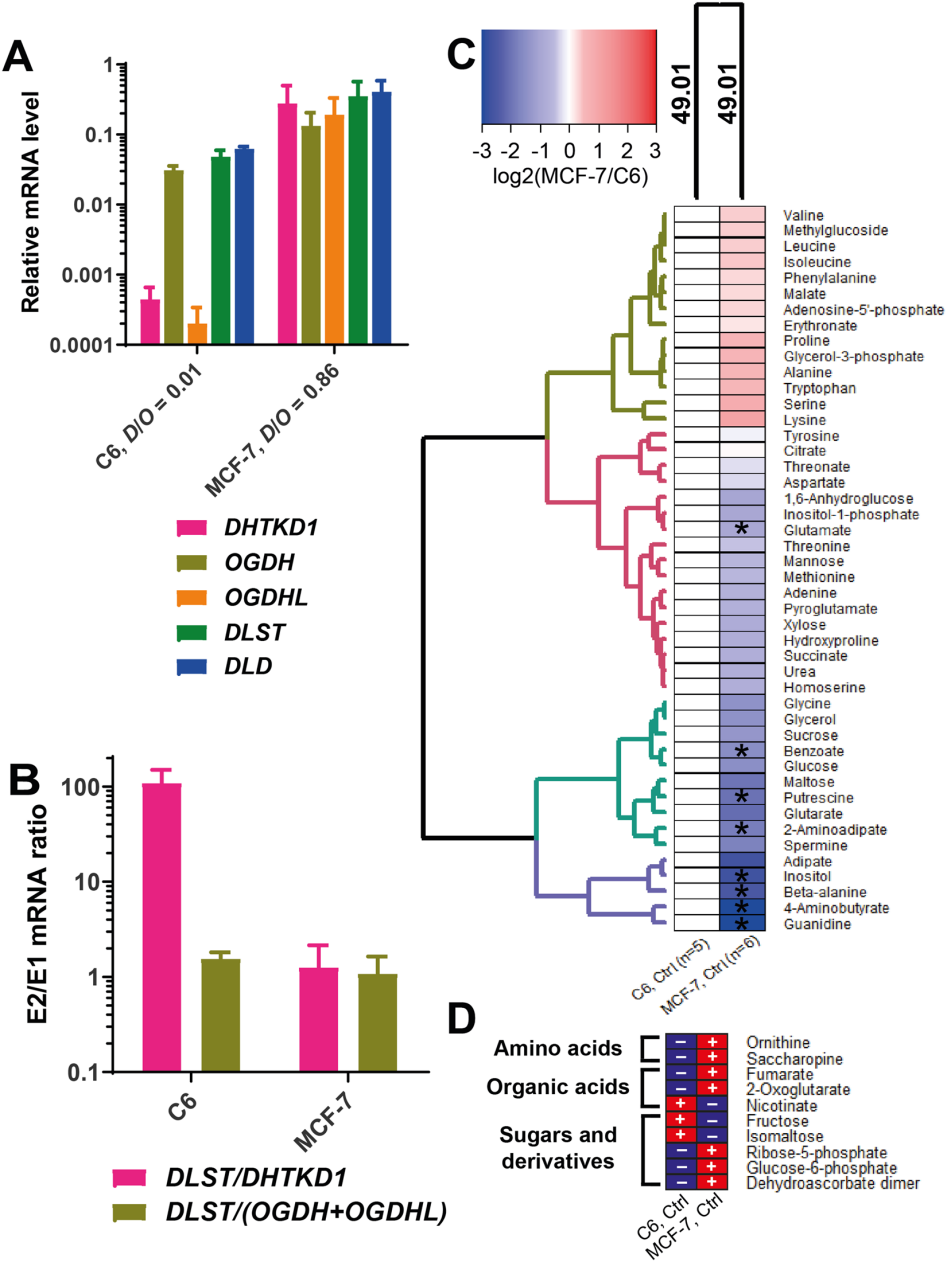
Cellular metabolomes at different expression of the OGDHC and OADHC components. (**A**) Relative mRNA levels for the enzymatic components of OGDHC and OADHC in C6 and MCF-7 cell lines. The shown transcript abundances are averaged from ≥8 independent experiments for each cell line. D/O is the ratio between the expression of *DHTKD1* and *OGDH* + *OGDHL* mRNAs. (**B**) Ratios of mRNA levels for the second and the first components of OADHC and OGDHC. (**C**) Relative metabolite abundances in the MCF-7 cell line compared to the C6 cell line. The asterisks show significant differences between the cell lines according to *t*-test. Experimental groups and metabolites are clustered using Manhattan as a distance measure and WPGMA as an agglomeration method. Number of independent experiments for each experimental group (n) is indicated at the bottom of the heatmap. (**D**) Cell-specific metabolites, i.e. those which are reliably quantified only in one of the two cell lines, are marked by “+” in the cell line where they are detected, and by “−” in the another one.

In view of cellular heterogeneity of mammalian tissues, metabolic significance of the *DHTKD1*-encoded OADH has been studied in cell lines which differ in OADH abundance. The C6 rat glioma and MCF-7 human breast adenocarcinoma cell lines (Fig. 5A) are similar to tissues with relatively low and high *DHTKD1* level, respectively (Fig. 2E). The critical ratios of *DLST* to *DHTKD1 vs DLST* to *OGDH(L)* also strongly differ in the C6 and MCF-7 lines (Fig. 5B), modelling the ratios existing in the tissues (Fig. 3B).

**Figure 6:**
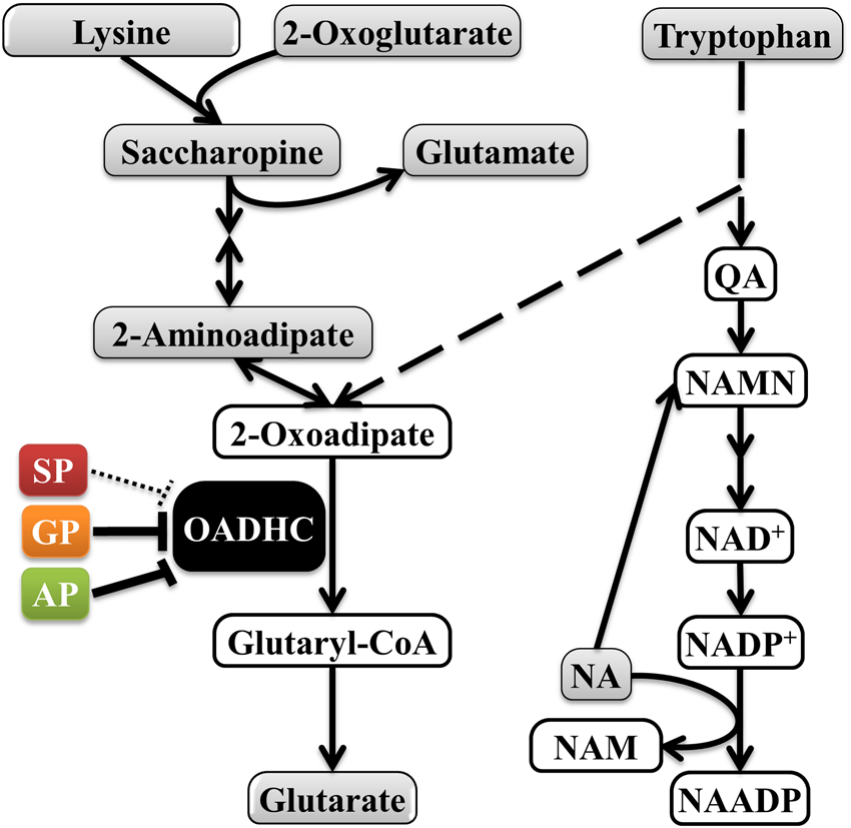
The OADHC-dependent pathways of the lysine and tryptophan catabolism, interacting with NAD metabolism. Lysine condenses with 2-oxoglutarate into saccharopine leading to 2-oxoadipate, whose oxidative decarboxylation by OADHC generates glutaryl-CoA. Glutarate is formed from glutaryl-CoA by hydrolysis or transacylation reactions^50^. Tryptophan is catabolised through alternative pathways leading to either 2-oxoadipate or quinolinic acid (QA). QA is an intermediate for the *de novo* biosynthesis of NAD from tryptophan^115,116^. In addition to its metabolic functions, NAD has signalling significance for glucose homeostasis, in particular through the Ca^2+^-mobilizing action of nicotinic acid adenine dinucleotide phosphate (NAADP), whose synthesis from NADP^+^ requires nicotinate. Dependent on cell-specific expression of the OADH-involving pathways, the OADH inhibition may downregulate saccharopine pathway and/or increase QA for biosynthesis of NAD and its signalling derivatives from tryptophan. The quantified metabolites of the presented pathways are shown in grey. Solid and dashed arrows indicate the single and more than two enzymatic reactions within a metabolic pathway, correspondingly. NA – nicotinic acid (nicotinate); NAADP – nicotinic acid adenine dinucleotide phosphate; NAM – nicotinamide; NAMN – nicotinic acid mononucleotide; QA – quinolinic acid.

The strong difference in the *DHTKD1* expression in the MCF-7 and C6 cells (Fig. 5A,B) is accompanied by the differences in cellular metabolomes (Fig. 5C,D). The 2-oxoadipate-linked metabolites (Fig. 6) are of special interest in this regard. Levels of lysine and tryptophan, whose degradation occurs through the common intermediate 2-oxoadipate, are proportional to the *DHTKD1* expression, which is higher in the MCF-7 cells, compared to the C6 cells. In contrast, the level of 2-aminoadipate is lower in MCF-7 *vs* C6 cells, in good accord with the known negative correlation between the level of 2-aminoadipate and the *DHTKD1* expression^27^. This finding confirms a higher flux of the oxidative decarboxylation of 2-oxoadipate in the cells with a higher *DHTKD1* expression (MCF-7). Association of the high *DHTKD1* expression with the saccharopine pathway of lysine catabolism (Fig. 6) is further supported by the high levels of 2-oxoglutarate and saccharopine in MCF-7 cells, whereas these metabolites are not detectable in C6 cells with a low expression of *DHTKD1* (Fig. 5D). In contrast, a metabolite of the NAD salvage pathway, nicotinate, is abundant in C6 cells and not detectable in MCF-7 cells (Fig. 5D). As shown in Fig. 6, nicotinate interacts with the tryptophan pathway of *de novo* NAD biosynthesis from quinolinic acid. Because this pathway is alternative to the tryptophan degradation to 2-oxoadipate, the low *DHTKD1* expression in C6 cells may increase the tryptophan flux to NAD synthesis (Fig. 6). As a result, the cell-specific upregulation of the saccharopine pathway is associated with upregulated expression of *DHTKD1*, whereas higher levels of nicotinate at a low expression of *DHTKD1* link OADH reaction and metabolism of NAD, which is also known to depend on tryptophan.

Remarkably, the differences in the 2-oxoadipate-linked metabolites are coupled to those in sugar metabolism: Compared to C6 cells, MCF-7 cells have more phosphorylated sugars and their derivatives (olive cluster at Fig. 5C,D), and less non-phosphorylated sugars (turquoise cluster at Fig. 5C,D). Besides, MCF-7 cells demonstrate higher levels of branched-chain amino acids, proline, serine, and ornithine, than C6 cells (olive cluster in Fig. 5C,D). Clusters of the metabolites, which are substantially lower in the MCF-7 relative to C6 cells (violet and turquoise clusters, Fig. 5C), include guanidine, polyamines, adipate and glutarate. Thus, the differences in the metabolomes of the cells with varied expression of *DHTKD1* reveal that the cell-specific involvement of OADHC in the lysine and tryptophan catabolic pathways is associated with altered metabolism of (phospho)sugars and guanidine/polyamine compounds.

### Effects of the phosphonate analogues of 2-oxodicarboxylic acids on metabolism of the cells with different *DHTKD1* expression

**Figure 7:**
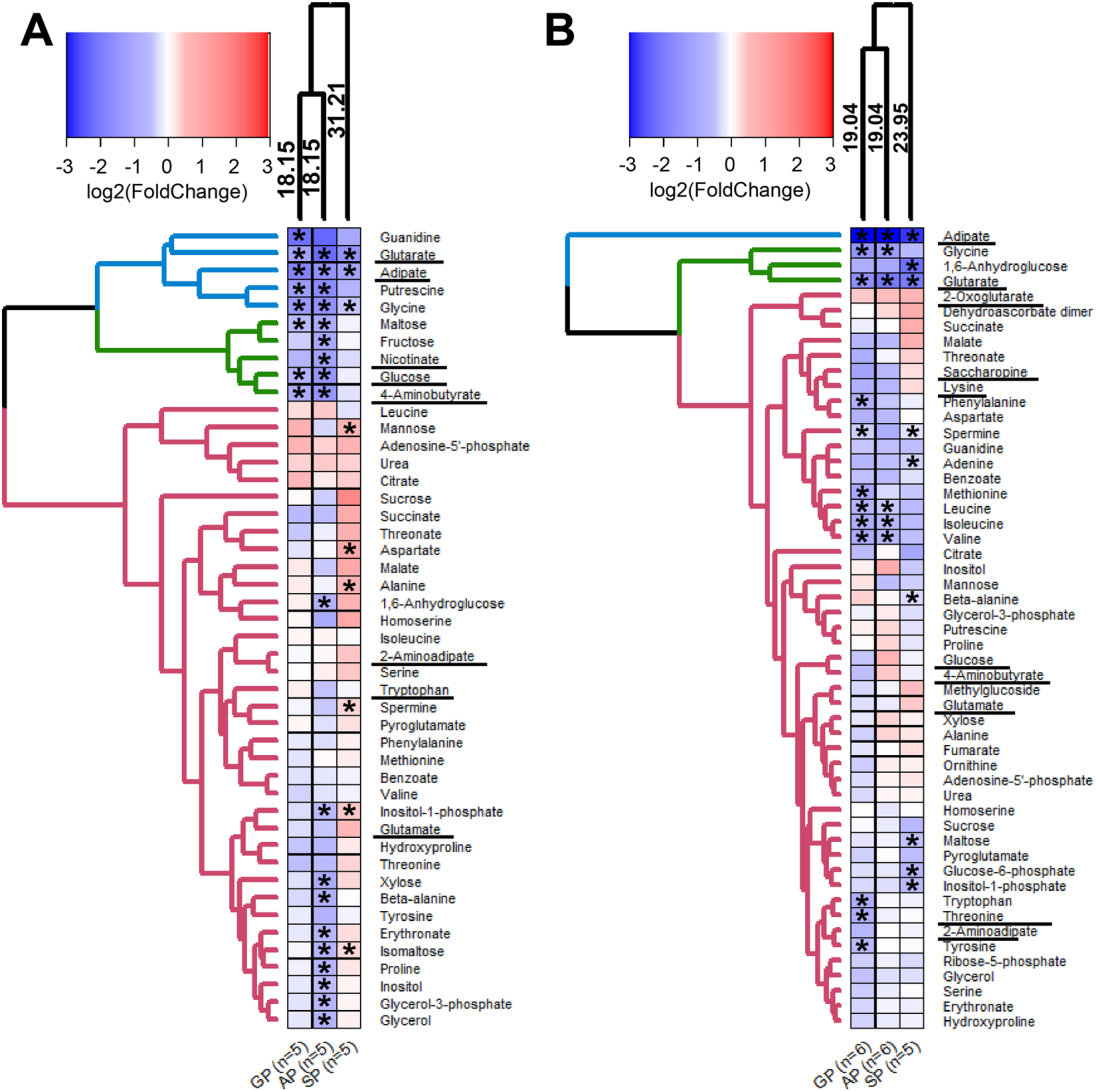
Effects of SP, GP and AP on cellular metabolomes. Metabolic perturbations in the C6 (**A**) and MCF-7 (**B**) cell lines after a 5 h incubation of the cells in minimal medium with succinyl phosphonate (SP), glutaryl phosphonate (GP) or adipoyl phosphonate (AP) (0.5 mM each) are shown as log_2_ of fold changes compared to the non-treated cells of the same batch. Clustering of experimental groups and metabolites uses Manhattan as a distance measure and WPGMA as an agglomeration method. Number of independent experiments for each experimental group (n) is indicated at the bottom of the heatmaps. The asterisks show significant (p ≤ 0.05) differences between the treated and non-treated cells according to *t*-test. The metabolites of the pathways discussed in the text, are underlined.

To characterize metabolic changes induced in the C6 and MCF-7 cells by the phosphonate inhibitors of OADHC and OGDHC, the cells were incubated for 5 h with a moderate (0.5 mM) concentrations of SP, GP or AP in the minimal medium. These conditions minimize cellular ability to compensate for the enzyme impairments by rearranging the metabolic processes due to the inhibition. Metabolic changes in the phosphonates-exposed cells point to the phosphonate-specific action on the overall metabolic profiles. As seen from Fig. 7A,B, the changes induced by SP are clustered separately from those caused by GP or AP. Thus, metabolic action of SP is different from that of either GP or AP. This finding on cell cultures is in good agreement with the preferred binding of SP to OGDHС and AP to OADHC *in vitro* (Fig. 4).

The calculated distances of the phosphonate-induced changes in the metabolic profiles of the cells indicate that the difference in the actions of SP *vs* GP or AP is higher in the C6 cells (31.21 *vs* 18.15, Fig. 7A), compared to the MCF-7 cells (23.95 *vs* 19.04, Fig. 7B). Moreover, the distance-defined specificity of the metabolic action of SP is more pronounced in C6 cells (31.21, Fig. 7A) with a lower *OGDH(L)* expression, than in MCF-7 cells (23.95, Fig. 7B) with a higher *OGDH(L)* expression (Fig. 5A). At the same time, the distances between the GP- or AP-induced metabolic changes are similar in both cell lines (18.15 in C6 *vs* 19.04 in MCF-7 cells). Thus, under equal experimental conditions, i.e. cellular incubation in minimal medium supplemented with the same concentration of the phosphonates, the metabolically different C6 and MCF-7 cells show different metabolic response to the phosphonates. The more pronounced metabolic effects of SP (C6 *vs* MCF-7 cells, Fig. 7) are observed at a lower abundance of *OGDH(L)* in the C6 *vs* MCF-7 cells (Fig. 5A), in accord with a lower spare OGDHC threshold capacity in C6 *vs* MCF-7 cells.

**Figure 8:**
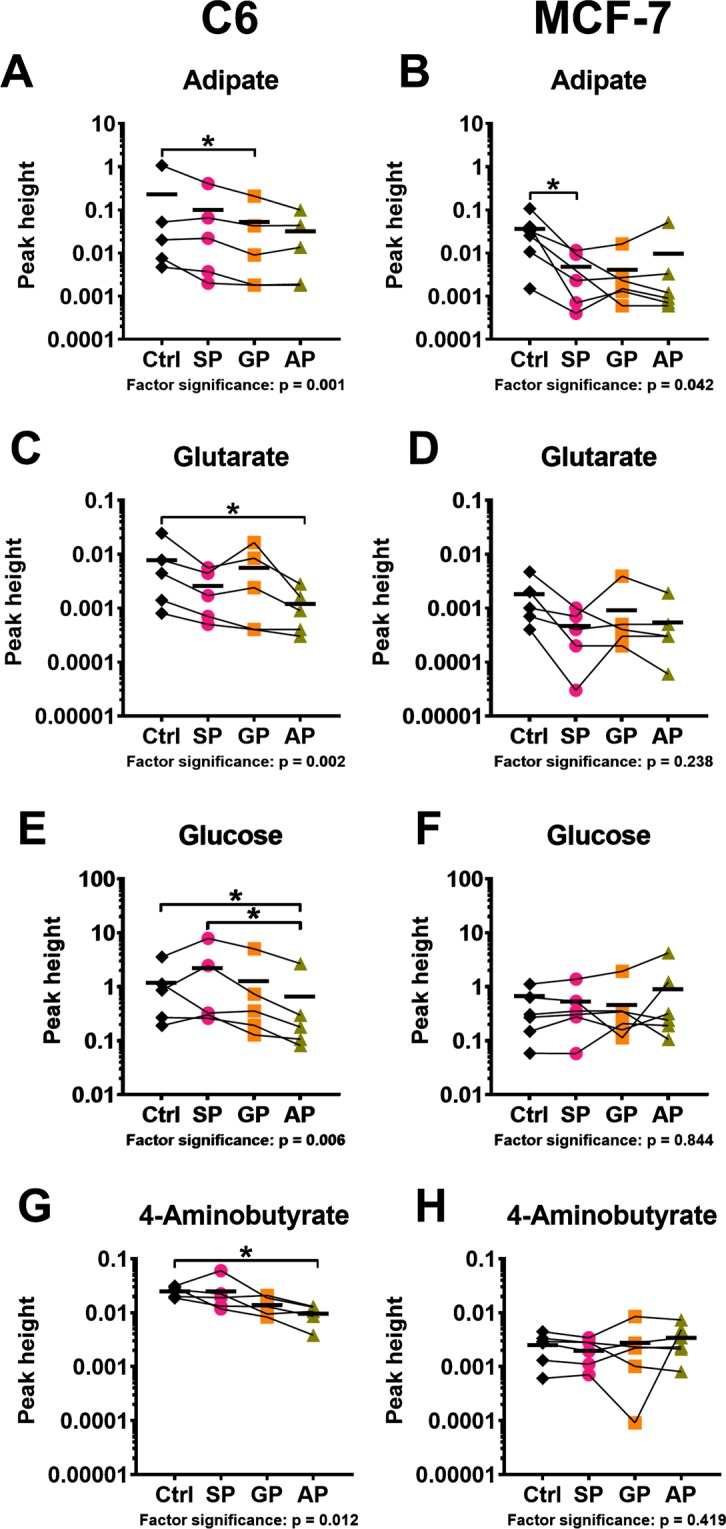
Comparison of the effects of SP, GP and AP on the levels of adipate (**A**,**B**), glutarate (**C**,**D**), glucose (**E**,**F**) and 4-aminobutyrate (**G**,**H**) in C6 (**A**,**C**,**E**,**G**) and MCF-7 (**B**,**D**,**F**,**H**) cells. Significances of the treatment factor according to Friedman’s test are indicated as p-values at the bottom of each graph. The asterisks show significant (p ≤ 0.05) differences between specific groups according to Dunn’s post-hoc test. Lines connect the values obtained in the same experiment. Mean values in the groups are shown by horizontal marks.

Full comparison of the four investigated groups to each other was done for selected metabolites, using their values normalized to internal standard and protein. Choice of the indicators, which were robustly determined in both cell lines, enabled additional comparison of the specificity of the phosphonates action, dependent on the metabolic type of a cell. As shown earlier, the studied cells demonstrated specific differences in the pathways (Fig. 6) involving nicotinate (detected only in C6 cells) or saccharopine (detected only in MCF-7 cells). Statistical significance in this comparison was assessed by Friedman’s test applicable for the data which do not have a normal distribution. The test relies not only on the average differences between the control and treated groups, but also on the consistency of changes in the repeated experiments. For instance, the mean values for adipate are reduced by both GP and AP in Fig. 8A, but AP reduces adipate only in the three out of five experiments, whereas GP does it in the four out of five experiments. Therefore, the GP action has a higher statistical significance according to Friedman’s test, despite the lower, compared to the GP treatment, mean level of adipate after the AP treatment (Fig. 8A). Likewise, statistical significance of the phosphonate-induced decreases in glutarate in MCF-7 cells (Fig. 8D) is lower, compared to that estimated by *t*-test (Fig. 7B), because the changes in separate experiments are not well coinciding. However, the finding of more profound AP-induced changes in C6 *vs* MCF-7 cells is supported by both the *t*-test (Fig. 7) and Friedman’s (Fig. 8) analyses. As seen from the p-values characterizing significance of the changes in the cells treated with the phosphonates, C6 cells are affected by the treatment much more than MCF-7 cells. The group comparison in each cell line reveals specific action of the phosphonates on glutarate, adipate, glucose and 4-aminobutyrate (Fig. 8). The significance of the adipate reduction, which is pronounced in both C6 and MCF-7 cells, is higher after the treatments with the OGDHC-targeting inhibitors SP and GP (Fig. 8A,B). Hence, inhibition of OGDHC is primarily responsible for the decrease in adipate, in good agreement with earlier findings that OGDHC regulates the mitochondrial entry of fatty acids^47^, whose β-oxidation inversely correlates with the adipate levels^48,49^. In contrast to adipate, glutarate decreases significantly only in C6 cells (Fig. 8C,D), and the most pronounced change in glutarate is induced by the OADH-preferring AP (Fig. 8C). As shown in Fig. 6, the reduced generation of an OADHC product glutaryl-CoA may decrease glutarate, as the latter is generated from the former through non-enzymatic hydrolysis or the transacylation reaction with other acyl-CoA derivatives^50^. The most significant decreases in glutarate by AP, compared to those by SP and GP, confirm that the observed decreases in glutarate mainly originate from the inhibition of OADHC. Because the degree of the glutarate decrease should be inversely related to the spare OADHC capacity of the cells, lower expression of *DHTKD1* in C6 *vs* MCF-7 cells leads to significant perturbation in the glutarate levels only in C6 cells. Remarkably, in C6 cells, not only the level of glutarate, but also that of glucose shows the strongest decrease after the treatment with the OADH-preferring AP, compared to the treatments with SP or GP (Fig. 8E). Moreover, the treatment of C6 cells with AP also significantly decreases 4-aminobutyrate, known to be strongly associated with perturbed glucose metabolism through the neurotransmitter function of this metabolite^51–55^ (Fig. 8G). In contrast, neither glutarate, nor glucose, nor 4-aminobutyrate are significantly affected in MCF-7 cells by any of the phosphonates (Fig. 8D,F,H). The coupling of the AP-induced perturbations in the OADHC function and cellular glucose level is in good accord with the known association between the glucose homeostasis and *DHTKD1* expression and/or function^23,27,56^.

The results of statistical analysis of the phosphonates-induced changes in the selected metabolic indicators according to Friedman’s test (Fig. 8) are supported by the results of clustering procedure presented in Fig. 7. The clustering ascribes glucose and 4-aminobutyrate to the same cluster not only in C6 cells, where the changes in these metabolites are statistically significant (Fig. 7A), but also in MCF-7 cells, where the metabolites are not significantly changed (Fig. 7B). However, only in C6 cells the cluster of glucose with 4-aminobutyrate belongs to the larger one including glutarate (Fig. 7A), whereas in MCF-7 cells the clustering does not reveal a relationship between these metabolites (Fig. 7B). In this regard, clustering of the changes in the C6 cell-specific metabolite nicotinate deserves attention. As shown in Fig. 7A, the phosphonates-induced changes in nicotinate are associated with those in glucose and 4-aminobutyrate, forming a larger cluster including also other sugars, i.e. maltose and fructose (green cluster in Fig. 7A). All metabolites of this cluster experience the most pronounced changes under the action of the bulkier phosphonates, GP or AP, and are affected much less significantly by SP. In contrast, specific action of SP increases the TCA-cycle-associated metabolites alanine and aspartate^36,57^ significantly more than GP or AP do. Close positioning of the nicotinate-including cluster of sugars (green cluster in Fig. 7A) and the cluster including adipate and glutarate (blue cluster in Fig. 7A) is worth noting in view of the known interactions of nicotinate and DHTKD1 with regulation of pathways involving not only lipids, but also glucose^23,27,56,58–67^.

Analysis of the correlated changes of all the quantified metabolites across the four experimental groups within specific metabolic networks of C6 or MCF-7 cells (Fig. S3) also reveals that in C6 cells the levels of nicotinate are coupled to those of glutarate and adipate. Moreover, this type of analysis indicates that all these metabolites form the cluster with 2-aminoadipate in C6 cells (Fig. S3A). In MCF-7 cells, the 2-aminoadipate cluster involves sugars and glutamate, but is distant from the cluster including lysine and saccharopine. Nevertheless, the cluster of lysine and saccharopine in MCF-7 cells is close to the one including the indicators of functions of the dehydrogenases of 2-oxoadipate and 2-oxoglutarate, i.e. glutarate and adipate (Fig. S3B), correspondingly.

As a result, the overall (Figs. 7 and S3) and specific (Fig. 8) changes in cellular metabolomes, which are observed after the phosphonates treatment of the cells with different *DHTKD1* expression, reveal specific actions of the shortest (SP) and longest (AP) phosphonates targeting OGDHC and OADHC, respectively (Fig. 4). In the cells with a low DHTKD1 expression, significant impairment in the OADH function, manifested in reduced glutarate level, is associated with perturbed glucose homeostasis (Fig. 8). Detection of strong changes in metabolic profiles of cells in minimal medium, induced by the homologous phosphonates in accordance with their *in vitro* specificity to OADH and OGDH and affecting different groups of structurally unrelated metabolites, indicate that the phosphonates penetrate into the cells and interact with their expected enzyme targets.

### Effects of the phosphonate analogues of 2-oxoglutarate and 2-oxoadipate on cellular NAD(P)H:MTT reductase activity

**Figure 9:**
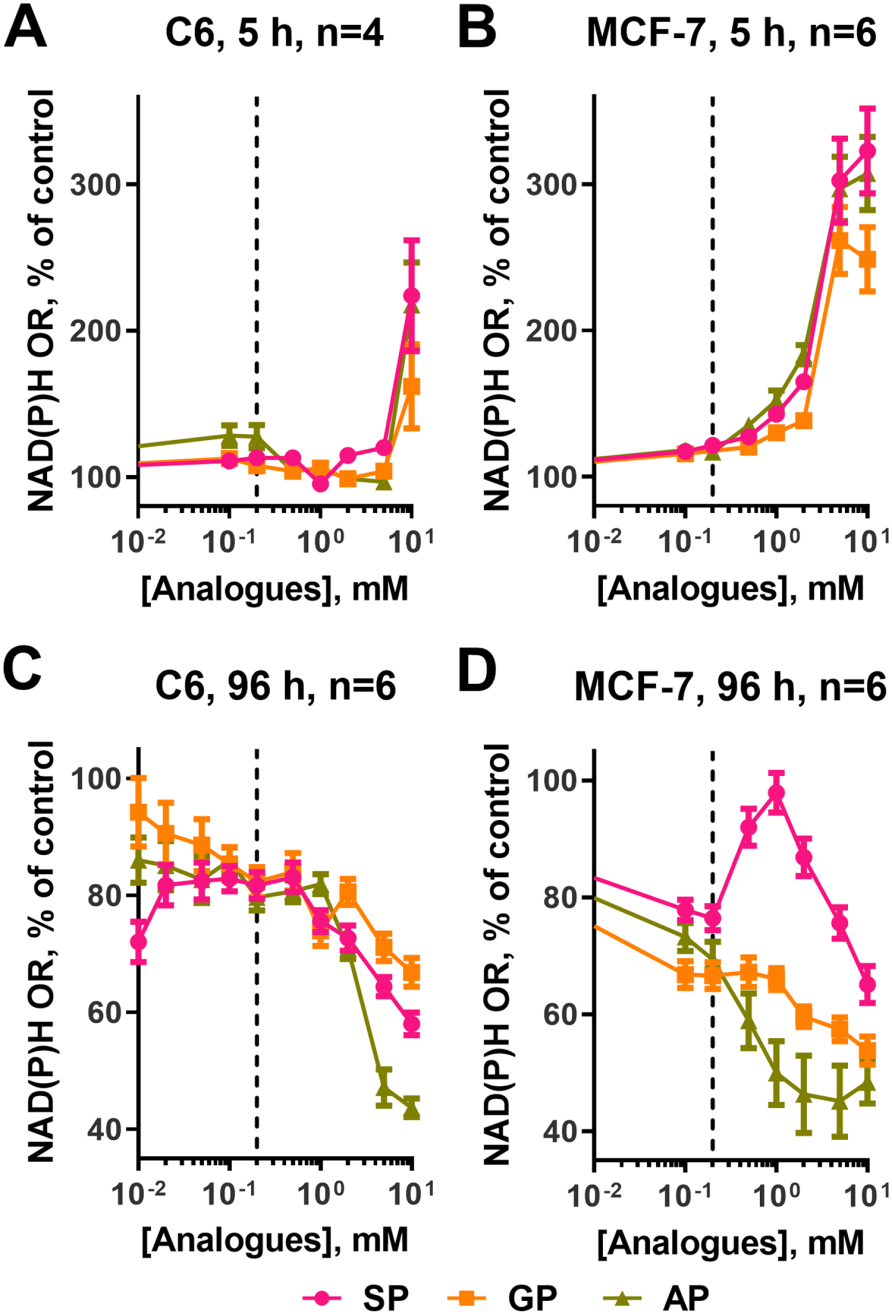
Comparison of the effects of SP, GP, and AP on cellular viability. Dependencies of NAD(P)H:MTT oxidoreductase activity of the C6 (**A**,**C**) and MCF-7 (**B**,**D**) cell lines on the concentrations of the phosphonates after a 5 h incubation in the minimal medium (**A**,**B**) or after a 96 h incubation in the optimal growth medium. (**C**,**D**) Data are presented as mean ± SEM.

Cellular reducing power, which could be assayed using the NAD(P)H-dependent reduction of artificial dyes, such as 3-(4,5-dimethylthiazol-2-yl)-2,5-diphenyltetrazolium (MTT), represents an integral indicator of cellular metabolism and proliferating ability. The changes in the reducing power of C6 and MCF-7 cells upon exposure to SP, GP or AP have been followed under the two sets of conditions: after a 5 h incubation with the analogues in a minimal medium (HBSS with glucose) and after a long (96 h) incubation in a complete growth medium (DMEM). The former conditions are the same as those where the cellular metabolic profiles presented in Fig. 7 have been assayed, whereas the latter conditions are used to assay the proliferating capacity of the cells. In addition to the action of the phosphonates on cellular metabolomes at a fixed concentration (Figs. 7 and 8), the concentration dependencies of cellular reducing power on the homologous phosphonates, which vary with the phosphonate structures (Fig. 9), point to intracellular action of the phosphonates on cellular metabolism.

A strong concentration-dependent increase in cellular NAD(P)H:MTT reductase activity after a 5 h treatment by all the phosphonates is higher in MCF-7 cells (3-fold) than in C6 cells (2-fold) (Fig. 9A,B). In addition to the higher amplitude, the increase in MCF-7 cells is observed at lower concentrations of the phosphonates (from 0.2 mM, dashed line in Fig. 9B), compared to C6 cells (at 10 mM, Fig. 9A). As a result, at 5 mM concentration of the analogues, C6 cells do not change their reducing power, whereas MCF-7 cells exhibit almost maximal increase. A higher increase in MCF-7 cells is also obvious after a 96 h incubation of the cells with the phosphonates (Fig. 9C,D). Under these conditions, it also becomes obvious that the increase in the cellular NAD(P)H:MTT reductase activity is much more pronounced after the treatment by SP, than GP or AP. In MCF-7 cells SP also causes the most pronounced decrease in the levels of adipate (Fig. 8B) manifesting increased β-oxidation of fatty acids. It is therefore probable that cellular NAD(P)H:MTT reductase activity in the SP-treated cells increases due to contribution of β-oxidation of fatty acids. The contribution may be increased upon impaired production of NADH in the TCA cycle whose flux is limited by the SP-inhibited OGDH^36^.

After a prolonged (96 h) incubation, cellular viability is strongly reduced in both cell lines by all the three phosphonates (Fig. 9C,D). Remarkably, the strongest antiproliferative action in both C6 and MCF-7 cells is exhibited by AP, whereas SP still induces compensatory changes manifested in temporary growth of cellular reducing power at 0.02–0.2 mM SP in C6 cells and 0.2–1 mM in MCF-7 cells (Fig. 9C,D). The data testify to more limitations for compensating perturbations in the OADH- than OGDH-dependent metabolic pathways in both C6 and MCF-7 cells.

## Discussion

Our study shows that OGDHC and OADHC extracted from mammalian tissues, are able to discriminate 2-oxoglutarate and 2-oxoadipate better than the complexes reconstructed from recombinant subunits^9,21^ do. While the recombinant complexes exhibit significant level of cross-reactivity to the 2-oxoglutarate and 2-oxoadipate^38^, the high selectivity to the cognate 2-oxo substrates is demonstrated for OADHC in the non-solubilized liver and spinal cord homogenates or for OGDHC in the solubilized heart homogenate (Fig. 2C,D), similar to our previous results on the complexes in the brain^68^. *In vivo*, the multienzyme organization of OADHC and OGDHC should be adjusted to the pathways where the complexes perform their specific biological roles. In this regard, the native organization of OADHC and OGDHC as separate molecular machines has more advantages than a hybrid complex incorporating different E1 components. Existence of the 2-oxo substrate-specific multienzyme structures is supported by different stability, localization and/or regulation of the OADHC and OGDHC-catalysed reactions, observed in this work and earlier upon animal supplementation with thiamine^68^ or mitochondrial production of reactive oxygen species^16^. Besides, the native structures may undergo different post-translational modifications and form heterologous complexes with other proteins of the specific metabolic pathways where OADHC and OGDHC participate. All these factors are known to affect the multienzyme structure of the 2-oxo acid dehydrogenase complexes^2^. The observed difference between the cross-reactivity to the 2-oxo substrates of the complexes present in animal tissues (this work) and those formed from recombinant components^9,21^, underlines significance of the study of native structures in addition to those assembled *in vitro* from the recombinant components.

New regulators of 2-oxo acid dehydrogenases have been developed in this work to specifically target the 2-oxo acid dehydrogenases isoenzymes transforming homologous substrates 2-oxoglutarate and 2-oxoadipate. Although polar molecules, such as 2-oxo phosphonates, do not diffuse across biological membranes, they may be transported by cellular carriers for similar compounds, such as 2-oxodicarboxylate carriers. In particular, slow transport of a phosphonate analogue of 2-oxoglutarate was suggested from the experiments with purified transporter of 2-oxoglutarate^36^. Intracellular action of the phosphonates is supported by the metabolic and viability effects of the phosphonates in cell lines (Figs. 7–9), correlating with *in vitro* specificity of their inhibitory action on OADHC and OGDHC (Fig. 4). Preferential inhibition of OGDHC by SP and OADHC by AP is revealed (Fig. 4) along with no significant effects of GP or AP on the enzymes transforming 2-oxo acids or their analogues are found (Table 1). Thus, confirming the previously established specificity of SP action *in vivo*^1,37,42,43^, the data of Table 1 show such a specificity for GP and AP as well. The specific binding of the 2-oxo substrates and phosphonates to the complexes *in vitro* agrees well with the results of cellular studies (Figs. 7–9), demonstrating that the metabolic and viability effects of the 2-oxoadipate mimics GP and AP strongly differ from those of the 2-oxoglutarate mimic SP (Figs. 7–9). Our recent structural data on the OGDH interaction with SP^69^ point to the role of specific catalysis-related conformational transitions in the strong inhibition exerted by the phosphonates on their cognate 2-oxo acid dehydrogenases complexes. In view of the competitive inhibition by 2-oxo phosphonates *vs* 2-oxo acids^2,37,69^, a much lower concentration of 2-oxoadipate *vs* 2-oxoglutarate in cells and tissues^11,70^ may increase the selectivity of the bulkier phosphonates GP and AP in targeting OADHC *vs* OGDHC. Thus, although one can never exclude an off-target effect of a drug, several lines of evidence provided in this work, strongly support the specific *in vivo* action of the phosphonate analogues of 2-oxoacids on the cognate dehydrogenases of 2-oxo acids. The supporting data include (i) screening of the enzymes from different classes and metabolic pathways regarding their reactivity to the phosphonates (Table 1), (ii) the metabolic actions of the homologous phosphonates (Figs. 7–9) in accordance with the preferential binding of SP to OGDHC and AP to OADHC (Fig. 4) and (iii) modelling of independent data on perturbed glucose metabolism in the *DHTKD1* mutants^23^ by AP better than by SP or GP (Fig. 8E).

The data obtained in this work pointed out the cell-specific involvement of OADHC not only into the saccharopine pathway, where the enzyme function has been studied earlier^24,27^, but also into metabolism of NAD. The two patterns of the *DHTKD1* expression in animal tissues and cells are revealed by our transcriptomics analysis (Figs. 2E and 5A). Cellular expression of *DHTKD1* is elevated along with the upregulated saccharopine pathway of lysine catabolism. Independent data confirm the prevalence of the saccharopine pathway in liver and kidney^71–76^, where the high level of *DHTKD1* expression is observed (Fig. 2). By contrast, the saccharopine pathway is downregulated during the brain development^71,74,77^, and the *DHTKD1* expression is not abundant in the adult rat brain (Fig. 2). However, a low OADH abundance, observed in most tissues (Fig. 2A), may be relevant for regulation of NAD metabolism (Fig. 6). Our data show that in the cells with a low expression of *DHTKD1*, the OADHC inhibitor AP changes the levels of both glucose and nicotinate, a NAD precursor (Fig. 7A). It is also known that NAD biosynthesis *de novo* occurs through the tryptophan transformation into quinolinic acid^78^ (QA, Fig. 6), which is alternative to the tryptophan catabolism generating 2-oxoadipate (Fig. 6)^78–81^. It may thus be suggested that the low abundance of OADH is involved in the regulation of the tryptophan fluxes through these alternative catabolic pathways. The OADH inhibition may increase the tryptophan flux to QA, thus elevating nicotinamide mononucleotide for biosynthesis of NAD (Fig. 6). This may alter the usage of cellular nicotinate in the pathway, increasing its involvement in biosynthesis of the signalling derivatives of NAD, such as nicotinic acid adenine dinucleotide phosphate and cyclic ADP-ribose phosphate^79,80^. Regulation of glucose homeostasis via the synthesis of these NAD derivatives^82–84^ from tryptophan is supported by the diagnostic value of plasma tryptophan levels in diabetes and obesity^85^, and altered *DHTKD1* expression under these pathologies^27,56,66,67^.

Thus, our study shows that incubation of C6 cells with a specific OADH inhibitor AP causes coupled changes in the levels of glucose, glutarate (the marker of OADH inhibition) and nicotinate (the marker of altered NAD metabolism). In addition to glucose, the levels of eight other sugars and their derivatives are significantly affected by AP in C6 cells (Fig. 7A). The findings support the AP-induced perturbations in sugar metabolism, coupled to alterations of NAD pathways.

As a result, the cell-specific features of the saccharopine pathway of lysine degradation or tryptophan-supported NAD biosynthesis may be addressed by different levels of *DHTKD1* expression. The present work demonstrates that the adipoyl and succinyl phosphonates have their preferred target enzymes, OADH and OGDH, respectively, and are able to discriminate metabolic contributions of their complexes in mammalian cells and tissues. Ability of adipoyl phosphonate to specifically regulate the *DHTKD1*-encoded protein *in vivo* may be useful to develop combinatorial therapies correcting pathological states associated with changed *DHTKD1* expression, among which are insulin resistance, obesity, and cancer.

## Materials and Methods

### Synthesis of the phosphonate analogues

All ^1^H, ^13^C and ^31^P NMR spectra were recorded at 400, 100.6 and 161.9 MHz, respectively with a Bruker Avance 400 spectrometer.

*Ethyl 4-(diethylphosphono)-4-oxobutanoate* (triethyl ester of succinyl phosphonate) and *trisodium 4-phosphono-4-*oxobutanoate (succinyl phosphonate, SP) were prepared as previously published^42^, using the Arbuzov reaction between triethyl phosphite and succinic acid monoethyl ester chloride (Reaction 5), followed by de-esterificatiozn with bromotrimethylsilane and NaOH (Reaction 6). The resulting trisodium salt of SP was 99% pure (with 1 mol. % of Na_2_HPO_4_ as impurity, based on NMR ^1^H and ^31^P spectra) and stable at room temperature as dry compound and at −20 °C as aqueous solution.

*Ethyl 5-(diethylphosphono)-5-oxopentanoate* (triethyl ester of glutaryl phosphonate). Triethyl phosphite (0.102 mol, 16.95 g, 17.5 mL) was added to glutaric acid monoethyl ester chloride (0.1 mol, 17.86 g, 15.7 mL) at 0 °C (Reaction 5). The resulting mixture was stirred at ambient temperature for 18 h. The product was isolated by vacuum distillation (b.p. 125–129 °C/0.9 mm, 20.43 g, 72.9% yield) as a colourless oil: NMR ^1^H (CDCl_3_), δ, ppm: 4.21 (dq, *J* 7.1 Hz, 1.1 Hz, 4 H, (CH_3_CH_2_O)_2_P(O)), 4.12 (q, *J* 7.1 Hz, 2 H, CH_3_CH_2_O), 2.91 (dt, *J* 7.1 Hz, 1.1 Hz, 2 H, CH_2_C(O)P(O)), 2.33 (t, *J* 7.2 Hz, 2 H, CH_2_COOEt), 1.94 (quint, *J* 7.2 Hz, 2 H, CH_2_CH_2_CH_2_), 1.36 (t, *J* 7.1 Hz, 6 H, (CH_3_CH_2_O)_2_P(O)), 1.24 (t, *J* 7.2 Hz, 3 H, CH_3_CH_2_O). NMR ^13^C (CDCl_3_), δ, ppm: 210.3 (d, *J* 167.8 Hz, C(O)P(O)), 172.5 (COOEt), 63.6 (d, *J* 7.6 Hz, (CH_3_CH_2_O)_2_P(O)), 60.2 (COOCH_2_CH_3_), 42.1 (d, *J* 54.8 Hz, CH_2_C(O)P(O)), 32.7 (CH_2_COOEt), 17.4 (CH_2_CH_2_CH_2_), 16.2 (d, *J* 5.9 Hz, (CH_3_CH_2_O)_2_P(O)), 14.0 (COOCH_2_CH_3_). NMR ^31^P (CDCl_3_), δ, ppm: −2.8.

*Trisodium 5-phosphono-5-oxopentanoate* (glutaryl phosphonate, GP). To ethyl 5-(diethylphosphono)-5-oxopentanoate (10 mmol, 2.8 g) stirred at 0 °C under argon, bromotrimethylsilane (40 mmol, 6.12 g, 5.3 mL) was added dropwise. The resulting solution was stirred at ambient temperature overnight. The excess of bromotrimethylsilane was removed under reduced pressure. 0.5 N NaOH (30.5 mmol, 61 mL) was added at 0 °C for Reaction 6, and the solution was stirred at 50 °C for 18 h. Water was evaporated to dryness under reduced pressure. Recrystallization from methanol followed by the recrystallization from aqueous ethanol gave the product as white solid (1.7 g, 64.9% yield) with 97% purity (with 3 mol. % of Na_2_HPO_4_ as impurity, based on NMR ^1^H and ^31^P spectra): NMR ^1^H (D_2_O), δ, ppm: 2.82 (t, *J* 7.4 Hz, 2 H, CH_2_C(O)P(O)), 2.19 (t, *J* 7.4 Hz, 2 H, CH_2_COOEt), 1.76 (quint, *J* 7.4 Hz, 2 H, CH_2_CH_2_CH_2_) (Fig. S2A). NMR ^13^C (D_2_O), δ, ppm: 229.3 (d, *J* 160.2 Hz, C(O)P(O)), 183.2 (COONa), 42.3 (d, *J* 41.3 Hz, CH_2_C(O)P(O)), 36.8 (CH_2_COONa), 19.5 (CH_2_CH_2_CH_2_) (Fig. S2B). NMR ^31^P (D_2_O), δ, ppm: −0.2 (Fig. S2C). Trisodium salt of GP was stable at room temperature as dry compound and at −20 °C as aqueous solution.

*Methyl 6-(dimethylphosphono)-6-oxohexanoate* (trimethyl ester of adipoyl phosphonate). Reaction 5 was performed using trimethyl phosphite (29.4 mmol, 3.65 g, 3.47 mL) added to methyl adipoyl chloride (28 mmol, 5 g, 4.36 mL) at 0 °C. The resulting mixture was stirred at ambient temperature for 18 h. The product was isolated by vacuum distillation (b.p. 123–125 °C/0.9 mm, 5.9 g, 83.9% yield) as colourless oil: NMR ^1^H (CDCl_3_), δ, ppm: 3.84 (d, *J* 10.7 Hz, 6 H, (CH_3_O)_2_P(O)), 3.65 (s, 3 H, CH_3_O), 2.83 (t, *J* 6.5 Hz, 2 H, CH_2_C(O)P(O)), 2.31 (t, *J* 6.5 Hz, 2 H, CH_2_COOMe), 1.64 (m, 4 H, CH_2_CH_2_CH_2_CH_2_), NMR ^13^C (CDCl_3_), δ, ppm: 210.0 (d, *J* 166.1 Hz, C(O)P(O)), 173.5 (COOMe), 53.8 (d, *J* 6.7 Hz, (CH_3_O)P(O)), 51.4 (COOCH_3_), 43.0 (d, *J* 54.8 Hz, CH_2_C(O)P(O)), 33.5 (CH_2_COOMe), 24.0 (CH_2_CH_2_C(O)P(O)), 21.6 (CH_2_CH_2_COOMe). NMR ^31^P (CDCl_3_), δ, ppm: −0.9.

*Trisodium 6-phosphono-6-oxohexanoate* (adipoyl phosphonate, AP). Bromotrimethylsilane (30 mmol, 4.59 g, 3.96 mL) was added dropwise to methyl 6-(dimethylphosphono)-6-oxohexanoate (8 mmol, 2.018 g) stirred at 0 °C under argon. The resulting solution was stirred at ambient temperature overnight. The excess of bromotrimethylsilane was removed under reduced pressure. 0.5 N NaOH (24 mmol, 48 mL) was added at 0 °C and the solution was stirred at ambient temperature overnight (Reaction 6). Water was evaporated to dryness under reduced pressure. Recrystallization from aqueous ethanol give the product as white solid (1.6 g, 72.5%) with 95% purity (with 5 mol. % of Na_2_HPO_4_ as impurity, based on NMR ^1^H and ^31^P spectra): NMR ^1^H (D_2_O), δ, ppm: 2.81 (t, *J* 6.7 Hz, 2 H, CH_2_C(O)P(O)), 2.18 (t, *J* 6.8 Hz, 2 H, CH_2_COOMe), 1.52 (m, 4 H, CH_2_CH_2_CH_2_CH_2_) (Fig. S2D). NMR ^13^C (D_2_O), δ, ppm: 230.1 (d, *J* 155.1 Hz, C(O)P(O)), 183.8 (COONa), 42.5 (d, *J* 40.5 Hz, CH_2_C(O)P(O)), 37.5 (CH_2_COONa), 25.5 (CH_2_CH_2_C(O)P(O)), 22.4 (CH_2_CH_2_COONa) (Fig. S2E). NMR ^31^P (D_2_O), δ, ppm: −0.1 (Fig. S2F). Trisodium salt of AP was stable at room temperature as dry compound and at −20 °C as aqueous solution.

### Enrichment of OGDHC and OADHC activities from animal tissues

Wistar male rats were used in the experiments to assess the effects of the phosphonates on the enzyme-enriched fractions from different tissues. All animal procedures were done according to Guide for the Care and Use of Laboratory Animals published by the US National Institutes of Health (NIH Publication No. 85–23, revised 1996) and the EU Directives 86/609/EEC and 2010/63/EU and approved by Bioethics Committee of Lomonosov Moscow State University. Animals were killed by decapitation.

Animal tissues for preparation of the OGDHC- and OADHC-enriched fractions were stored frozen at −70 °C. The homogenization and protein solubilization were done according to the published procedure of Stanley and Perham^86^, with some modifications. Except where indicated, all procedures were performed at 4 °C. Liver and heart samples were cut in small cubes and homogenized in double volume of isolation buffer (50 mM MOPS, 2.7 mM EDTA, 0.2 mM AEBSF, 0.16 µM aprotinin, 3.33 µM bestatin, 3 µM E-64, 2 µM leupeptin, and 1.4 µM pepstatin A, pH 6.8) using IKA T-10 Basic tissue disperser followed by several strokes in Potter-Elvehjem homogenizer. The homogenate was diluted twice with the same buffer, containing 6% Triton X-100 and with pH adjusted to 7.0, and centrifuged for 20 min at 18500 g. The pellet was washed with isolation buffer (2.5 volumes of initial tissue) containing 3% Triton X-100 and with pH adjusted to 7.0 and centrifuged as above. The supernatants from two centrifugations were combined and made 1 µg/mL with cytochalasin D. After 10 min pH was lowered to 6.4 with 10% acetic acid, 0.12 volumes of 35% polyethylene glycol (PEG)-6000 were added, followed by 30 min stirring and centrifugation for 20 min at 18500 g. The resulting pellet was resuspended in isolation buffer (1.6 volumes of initial tissue) using Potter-Elvehjem homogenizer, sonicated for 30 s in Branson 2510 water bath, and clarified for 40 min at 20 °C and 18500 g. The supernatant was cleared from excessive fat using four layers of cheesecloth, pH was adjusted to 7.0 with 0.5 M KOH followed by addition of 13 mM MgCl_2_. After 5 min incubation at 30 °C and cooling down the ionic strength of the suspension was increased by addition of 1 M potassium phosphate buffer (pH 6.3) up to 50 mM concentration. Similar to previous precipitation with PEG, 1 µg/mL cytochalasin D was added, followed by pH adjusting to 6.4 with 10% acetic acid, addition of 0.12 volumes of 35% PEG-6000 were added, 30 min stirring and centrifugation for 20 min at 18500 g. The pellet was resuspended in storage (0.4–0.6 volumes of initial tissue) buffer (50 mM KH_2_PO_4_, 20% glycerol, 0.1 mM EDTA, 1 mM MgCl_2_, 1 mM ThDP, 0.2 mM AEBSF, 0.16 µM aprotinin, 3.33 µM bestatin, 3 µM E-64, 2 µM leupeptin, and 1.4 µM pepstatin A, pH 6.8) using Potter-Elvehjem homogenizer.

After standing overnight, the pellets were briefly sonicated and clarified by centrifugation at room temperature. The resulting samples were used for kinetic assays and stored on ice at 4 °C with app. 20–30% OGDHC and OADHC inactivation within two weeks. The heart preparations also contained the activity of pyruvate dehydrogenase complex and liver preparation – the activity of branched-chain 2-oxo acid dehydrogenase complex, which were utilized in the studying of the specificity of the inhibitory effects of 2-oxo phosphonates.

Protein concentration in fractions was measured using Bradford assay^87^ with modification of Zor & Selinger^88^.

### Tissue homogenization for enzyme activity measurement and Western blotting

Tissue samples were homogenized in 50 mM MOPS, pH = 7.0, 20% glycerol and 2.7 mM EDTA in IKA T10 basic homogenizer. Sonication and detergent extraction of the homogenates was performed as previously described for the rat brain homogenates^68^. For Western blotting, the non-sonicated homogenates were diluted to app. 8.5 mg tissue per mL, and proteins were separated on hand-cast 10% acrylamide gels containing 0.5% 2,2,2-trichloroethanol. For detection of the *DHTKD1*-encoded protein, gels were blotted onto PVDF membrane using PowerBlotter (Thermo Scientific) and probed with anti-DHTKD1 antibodies (PA5–24208, Invitrogen, 1:1000) and HRP-conjugated secondary antibodies (P-GAM Iss, Imtek, Russia, 1:1000) in iBind Western Device (Thermo Scientific). Proteins were visualized using Clarity Western ECL substrate (Bio-Rad) and ChemiDoc MP Imaging System (Bio-Rad). Band intensities were calculated using ImageLab software (Bio-Rad) and normalized by relative whole protein level, measured as combined signal in the 20–250 kDa region of the corresponding gel lane using stain-free gel mode in ChemiDoc MP and ImageLab. Raw images of anti-DHTKD1-stained membrane and of total protein visualized in gel are given in Fig. S1A,B, respectively.

### Protein detection by mass-spectrometry

Crude mitochondria-enriched fractions, prepared from fresh rat livers and cerebral cortices were obtained according to published procedures^4,89^. The proteins were separated in polyacrylamide gels and subjected to in-gel digestion by trypsin and analysed by LC-MS/MS as described earlier^90^. Protein identification was performed by PEAKS Studio 8.0 (Bioinformatics Solutions) with false discovery rate set to <1%. The number of identified peptides for DHTKD1, OGDH or DLST proteins was used as a semi-quantitative measure of their expression, as previously described^90^, since the two variables roughly correlate with each other^4^.

### Enzyme activity assays

Enzyme activities of OADHC and OGDHC in tissue homogenates and enriched fractions were assayed in the overall reactions (Fig. 1B, Reaction 4) by spectrophotometric quantification of NADH production at 340 nm using Sunrise microplate reader (Tecan) and molar extinction coefficient of NADH 6220 M^−1^*cm^−1^, as previously described^68^. The assay medium (pH 7.0) contained 50 mM MOPS, 1 mM DTT, 1 mM MgCl_2_, 1 mM CaCl_2_, 1 mM ThDP, 2.5 mM NAD^+^, 0.05 mM CoASH, and 2 mM 2-oxoglutarate or 2-oxoadipate, if not specified otherwise. Initial rates of the OGDH and OADH reactions, catalysed by the tissue homogenates, were determined during 5 min. For the enzyme-enriched preparations, the time interval between 3 and 10 min from the reaction start, was found optimal, as the burst kinetics of the blank reaction lacking the 2-oxo substrate, was completed within 3 min. All the reaction rates were corrected for the rates of the blank reactions in the medium omitting 2-oxo acid substrate. Activities of other enzymes related to 2-oxo acid metabolism, were assayed in the following media: pyruvate dehydrogenase complex – in 50 mM MOPS (pH 7.8) containing 1 mM DTT, 1 mM MgCl_2_, 1 mM CaCl_2_, 1 mM ThDP, 2.5 mM NAD^+^, 0.05 mM CoASH, and 0.2 mM pyruvate; branched-chain 2-oxo acid dehydrogenase complex – in 50 mM MOPS (pH 7.4) containing 1 mM DTT, 1 mM MgCl_2_, 1 mM CaCl_2_, 1 mM ThDP, 2.5 mM NAD^+^, 0.05 mM CoASH, and 0.2 mM 3-methyl-2-oxovalerate; glutamate dehydrogenase – in 0.1 M Tris-HCl (pH 7.5) containing 0.2 mM NADH, 50 mM NH_4_Cl, 0.1 mM ADP, and 0.2 mM 2-OG; malate dehydrogenase – in 0.1 M Tris-HCl (pH 7.5) containing 0.14 mM NADH and 0.2 mM oxaloacetate; lactate dehydrogenase – in 0.1 M Tris-HCl (pH 7.3) containing 0.15 mM NADH and 0.2 mM pyruvate; pyruvate kinase – in 0.1 M Tris-HCl (pH 7.5) containing 0.22 mM NADH, 1.5 mM ADP, 20 mM KCl, 60 mM MgCl_2_, 1.5 µg/mL lactate dehydrogenase and 0.2 mM phosphoenolpyruvate; glutamate oxaloacetate transaminase – in 0.1 M potassium phosphate buffer (pH 7.4 adjusted with NaOH) containing 40 mM D,L-aspartate, 0.1 mM NADH, 5 µg/mL malate dehydrogenase and 2 mM 2-oxoglutarate; glutamate pyruvate transaminase – in 0.1 M potassium phosphate buffer (pH 7.4 adjusted with NaOH) containing 40 mM D,L-alanine, 0.1 mM NADH, 5 µg/mL lactate dehydrogenase and 2 mM 2-oxoglutarate; NADP^+^-dependent isocitrate dehydrogenase – in 50 mM Tris-HCl (pH 7.5) containing 0.5 mM NADP^+^, 3.5 mM MgCl_2_, and 2 mM isocitrate; NADP^+^-dependent malic enzyme – in 0.1 M Tris-HCl (pH 7.5) containing 0.4 mM NADP^+^, 4 mM MgCl_2_, and 2 mM malate.

Activities of the pyruvate and branched-chain 2-oxo acid dehydrogenase complexes were measured in enzyme-enriched preparations, obtained from the rat heart and liver as described above. Other enzymes were assayed in sonicated and detergent-treated homogenates of the same tissues and/or commercially available purified enzymes. All reaction rates were measured at stationary phases and corrected for background reactions in the media omitting the 2-oxo acid substrates or their analogues.

### Cellular studies

Dulbecco’s modified Eagle’s medium (DMEM; Sigma D6046, supplemented with 10% FBS, 100 IU/mL penicillin and 100 µg/mL streptomycin) and DMEM/F12 1:1 (Sigma D8437, supplemented with the same reagents) media were used for culturing C6 rat glioma and MCF-7 human breast adenocarcinoma cell lines, respectively. Both lines were obtained from American Type Culture Collection (LGC Standards, Poland). To grow cells for the viability tests, the cell-optimized densities were used: C6 cells were seeded in 96-well plates in 100 µl DMEM at a density of 5*10^3^ cells/well for 5 h incubation or 1*10^3^ cells/well for 96 h incubation; MCF-7 cells were seeded in 100 µl DMEM/F12 at a density of 1.5*10^4^ cells/well for 5 h incubation or 5*10^3^ cells/well for 96 h incubation. After 24 h the media were exchanged for HBSS, supplemented with 1 g/L glucose, 1.3 mM CaCl_2_, 1.0 mM MgSO_4_, and phosphonate analogues (0–10 mM) for 5 h incubation, as previously described^91,92^. For 96 h incubation, the media were exchanged for the same ones including the phosphonates. After 5 or 96 h incubation with the analogues, changes in the NAD(P)H levels were measured by using the redox indicator, 3-(4,5-dimethylthiazol-2-yl)-2,5-diphenyltetrazolium, as previously described^92^, with sodium dodecyl sulphate used as a solvent for formazan crystals.

For measuring the levels of different metabolites, cells were seeded on 5 or 10 mL Petri dishes (5*10^5^ or 1.5*10^6^ cells/dish for C6 cells; 1.5*10^6^ or 4*10^6^ cells/dish for MCF-7 cells) in the same growth medium as described above for viability tests. 24 h later, the medium was exchanged for HBSS, supplemented with 1 g/L glucose, 1.3 mM CaCl_2_, and 1.0 mM MgSO_4_. The control cells and cells with 0.5 mM of SP, GP or AP added to the medium were further incubated for 5 h. After that, the cells were washed twice with PBS, and metabolites were extracted for quantification of metabolite abundance. 2 mL of ice-cold methanol containing 50 μM ribitol as internal standard^93^ were used for extraction of cells grown on 5 mL Petri dishes; for cells grown on 10 mL dishes, 4 mL of the extracting reagent were used instead. The extracts were centrifuged for 20 min at 65536 g, 4 °C, with the supernatants and pellets used for the quantification of metabolites and total protein, correspondingly, after storage at −70 °C.

### Analysis of the transcriptomic and proteomic data on the enzymes of interest

Transcriptomic data representing the results of the microarray and RNA-Seq experiments were obtained from the NCBI Gene Expression Omnibus database^94,95^. The data from several independent experiments are presented as mean ± standard error of mean (SEM). Signals from various samples corresponding to the full-length mRNA transcripts of the genes of interest were normalized as described previously^96^, using the sum of the levels of the two abundant housekeeping genes, i.e. *GAPDH* and *ACTB*, for the normalization.

Data on the tissue expression of the enzymes of interest were taken from the following series of experiments: GSE47792 (experiments GSM1328669-1328672 and GSM1328685-1328688), GSE63362 (experiments GSM1547410-1547414), and GSE13271 (experiments GSM334951-334955 and GSM334961-334965) for the rat skeletal muscles (gastrocnemius, soleus or without the muscle specification); GSE47792 (experiments GSM1328541-1328544 and GSM1328557-1328560), GSE63362 (experiments GSM1547360-1547366), and GSE30308 (experiments GSM751257-751261) for the rat heart; GSE43013 (experiments GSM1055075-1055075), GSE47792 (experiments GSM1328573-1328576 and GSM1328589-1328592), GSE63362 (experiments GSM1547372-1547381), and GSE8438 (experiments GSM1411057-1411061) for the rat kidney; GSE43013 (experiments GSM1055025-1055027), GSE47792 (experiments GSM1328637-1328640 and GSM1328653-1328656), GSE63362 (experiments GSM1547382-1547390), and GSE13271 (experiments GSM335052-335056 and GSM335062-335066) for the rat liver; GSE93249 (experiments GSM2449152-2449154), GSE88855 (experiment GSM2350926), GSE69334 (experiments GSM1698021-1698023), GSE29488 (experiments GSM729611-729613), GSE46988 (experiments GSM1142632-1142635), and GSE67830 (experiments GSM1656755-1656758) for rat spinal cord; GSE43013 (experiments GSM1055120-1055122), GSE47792 (experiments GSM1328509-1328512 and GSM1328525-1328528), GSE63362 (experiments GSM1547288-1547297), and GSE64617 (experiments GSM1575587-1575596) for the rat brain; GSE44786 (experiments GSM1091761-1091763), GSE96085 (experiments GSM2532938-GSM2532943), GSE68973 (experiments GSM1689113-1689117), and GSE32432 (experiments GSM802597-802598 and GSM802604-802605) for rat mammary gland.

Data on the expression of the enzymes of interest in cell cultures were taken from the following series of experiments: GSE89873 (experiments GSM2392321-2392326) and GSE47529 (experiments GSM1151629-1151630 and GSM1151643-1151646) for C6 cell line; GSE75570 (experiments GSM1959233-1959235), GSE26537 (experiments GSM652402-652404), GSE6462 (experiments GSM148516-148517), GSE52674 (experiments GSM1273928-1273929), GSE6521 (experiments GSM149913-149914), GSE8471 (experiment GSM210180), GSE13009 (experiment GSM325937), and GSE21618 (experiment GSM539725), together with data from three RNA-Seq experiments from the EMBL Expression Atlas database^75,76^ for the MCF-7 cell line.

Proteomics data originating from the mass spectrometry experiments were obtained from EMBL Expression Atlas^75,76^, PaxDB^97,98^ and MOPED^99,100^ databases, containing the data taken from several research articles. Due to the absence of the high-throughput proteomic studies for the examined rat tissues, the data obtained in mice were used instead. Protein expression in the murine skeletal muscles^101,102^, heart^101,103–105^, kidney^101,104^, liver^101,103,104,106,107^, spinal cord^108^ and brain^101,103,104,109^ was assessed. The extracted signals, expressed as parts per million, correspond to values, normalized to total peptide counts. The data on specific tissues are presented as mean ± SEM of several independent experiments.

### Metabolic profiling

Metabolic profiling in methanol cell extracts was done as previously described^92,110^. Briefly, the frozen extracts were thawed, separated into polar and non-polar phases in a mixture of chloroform and water by centrifugation, after which the 0.3 mL aliquots of polar phase were vacuum-dried. 40 μL of 20 mg/mL methoxamine hydrochloride in pyridine was added to the dried residue to protect reactive oxo groups in metabolites during derivatization. After incubation for 2 h at 37 °C, 68.6 μL of N-methyl-N-(trimethylsilyl)trifluoroacetamide (for derivatization of polar groups) and 1.4 μL of the mixture of the methyl esters of various fatty acids (for calibration of the chromatograph) were added to the extracts, and the samples were derivatized for 30 min at 37 °C.

After the incubation, the samples were analysed using a gas chromatography-coupled mass spectrometer Pegasus BT (LECO). Chromatograms and mass spectra were analysed using the ChromaTOF (LECO) and OpenChrom (open-source) software. TagFinder software^111^ together with the metabolite library of Max Plank Institute of Molecular Plant Physiology^112,113^ were used for the peak annotation and quantification. Only the metabolites which were reliably detected in all experiments, were used for characterization of the phosphonate effects. The metabolite peak heights were normalized for that of ribitol and for total protein content, measured by the Bradford assay^87^ in the cell pellet, obtained during methanol extraction procedure.

Influence of a phosphonate on metabolic profile in each experiment was estimated by normalization of the data obtained from the phosphonate-treated cells to the control cells in the same experiment. The ratios obtained from independent experiments were log2-transformed and used to find mean ± SEM of the changes in metabolic profiles. For comparison between non-treated C6 and MCF-7 cell lines, the mean values were determined from log2-transformed absolute levels. Heatmaps were built in RStudio 1.2 (RStudio Inc.).

### Statistical analysis

Fold changes in metabolite abundances were calculated within one cell batch, log2-transformed and averaged using the data obtained with different cell batches. One-sided unpaired *t*-test was applied to the log2-transformed ratios to reveal statistical significance of differences between the phosphonate-treated and control cells. To compare the untreated C6 and MCF-7 cell lines, the unpaired *t*-test was applied to ribitol- and protein-normalized and log2-transformed peak heights.

Multiple groups were compared either by one-way ANOVA or Friedman’s tests, followed by Tukey’s or Dunn’s post-hoc tests, respectively, depending on the results of Shapiro-Wilk test for normality. The specific details of the analysis are indicated in the figure captions. For visualization of correlation matrices, Spearman’s correlation coefficients and their p-values were calculated. Differences and correlations with p ≤ 0.05 were considered significant.

Standard deviation (SD) for the ratios was calculated as previously described^114^. SEM for the ratios was calculated via dividing the resulting SD by the square root of sum of sample numbers.

## Supplementary information

Supplementary information.

## References

[1] Artiukhov AV, Bunik VI, Graf AV (2016). Directed regulation of multienzyme complexes of the 2-oxo acid dehydrogenases using phosphonate and phosphinate analogs of 2-oxo acids. Biochemistry. Biokhimiia.

[2] Bunik, V. *Vitamin-dependent complexes of 2-oxo acid dehydrogenases: structure*, *function*, *regulation and medical implications*. (Nova Science Publishers, 2017).

[3] Bunik VI, Degtyarev D (2008). Structure-function relationships in the 2-oxo acid dehydrogenase family: substrate-specific signatures and functional predictions for the 2-oxoglutarate dehydrogenase-like proteins. Proteins.

[4] Bunik V, Kaehne T, Degtyarev D, Shcherbakova T, Reiser G (2008). Novel isoenzyme of 2-oxoglutarate dehydrogenase is identified in brain, but not in heart. The FEBS journal.

[5] Hoque MO (2008). Genome-wide promoter analysis uncovers portions of the cancer methylome. Cancer research.

[6] Ostrow KL (2009). Pharmacologic unmasking of epigenetically silenced genes in breast cancer. Clinical cancer research: an official journal of the American Association for Cancer Research.

[7] Sen T (2012). OGDHL is a modifier of AKT-dependent signaling and NF-kappaB function. PloS one.

[8] Fedorova MS (2015). [Downregulation of OGDHL expression is associated with promoter hypermethylation in colorectal cancer]. Molekuliarnaia biologiia.

[9] Nemeria NS (2018). The mitochondrial 2-oxoadipate and 2-oxoglutarate dehydrogenase complexes share their E2 and E3 components for their function and both generate reactive oxygen species. Free radical biology & medicine.

[10] Sherrill, J. D. *et al*. Whole-exome sequencing uncovers oxidoreductases DHTKD1 and OGDHL as linkers between mitochondrial dysfunction and eosinophilic esophagitis. *JCI insight* **3**, 10.1172/jci.insight.99922 (2018). 10.1172/jci.insight.99922 PMC5931135 29669943

[11] Danhauser K (2012). DHTKD1 mutations cause 2-aminoadipic and 2-oxoadipic aciduria. American journal of human genetics.

[12] Hagen J (2015). Genetic basis of alpha-aminoadipic and alpha-ketoadipic aciduria. Journal of inherited metabolic disease.

[13] Stiles Ashlee R., Venturoni Leah, Mucci Grace, Elbalalesy Naser, Woontner Michael, Goodman Stephen, Abdenur Jose E. (2015). New Cases of DHTKD1 Mutations in Patients with 2-Ketoadipic Aciduria. JIMD Reports.

[14] Bunik VI, Pavlova OG (1993). Inactivation of alpha-ketoglutarate dehydrogenase during oxidative decarboxylation of alpha-ketoadipic acid. FEBS letters.

[15] Bunik V, Westphal AH, de Kok A (2000). Kinetic properties of the 2-oxoglutarate dehydrogenase complex from Azotobacter vinelandii evidence for the formation of a precatalytic complex with 2-oxoglutarate. European journal of biochemistry/FEBS.

[16] Goncalves RL, Bunik VI, Brand MD (2016). Production of superoxide/hydrogen peroxide by the mitochondrial 2-oxoadipate dehydrogenase complex. Free radical biology & medicine.

[17] Wong HS, Dighe PA, Mezera V, Monternier PA, Brand MD (2017). Production of superoxide and hydrogen peroxide from specific mitochondrial sites under different bioenergetic conditions. The Journal of biological chemistry.

[18] Habarou F (2017). Biallelic Mutations in LIPT2 Cause a Mitochondrial Lipoylation Defect Associated with Severe Neonatal Encephalopathy. American journal of human genetics.

[19] Mayr JA, Feichtinger RG, Tort F, Ribes A, Sperl W (2014). Lipoic acid biosynthesis defects. Journal of inherited metabolic disease.

[20] Tort F, Ferrer-Cortes X, Ribes A (2016). Differential diagnosis of lipoic acid synthesis defects. Journal of inherited metabolic disease.

[21] Nemeria NS (2017). The human Krebs cycle 2-oxoglutarate dehydrogenase complex creates an additional source of superoxide/hydrogen peroxide from 2-oxoadipate as alternative substrate. Free radical biology & medicine.

[22] Xu WY (2012). A nonsense mutation in DHTKD1 causes Charcot-Marie-Tooth disease type 2 in a large Chinese pedigree. American journal of human genetics.

[23] Xu, W. Y. *et al*. DHTKD1 Deficiency Causes Charcot-Marie-Tooth Disease in Mice. *Molecular and cellular biology* **38**, 10.1128/MCB.00085-18 (2018). 10.1128/MCB.00085-18 PMC6002691 29661920

[24] Biagosch C (2017). Elevated glutaric acid levels in Dhtkd1-/Gcdh- double knockout mice challenge our current understanding of lysine metabolism. Biochimica et biophysica acta.

[25] Bansagi B (2017). Genetic heterogeneity of motor neuropathies. Neurology.

[26] Wang TJ (2013). 2-Aminoadipic acid is a biomarker for diabetes risk. The Journal of clinical investigation.

[27] Wu Y (2014). Multilayered genetic and omics dissection of mitochondrial activity in a mouse reference population. Cell.

[28] Sell DR, Strauch CM, Shen W, Monnier VM (2007). 2-aminoadipic acid is a marker of protein carbonyl oxidation in the aging human skin: effects of diabetes, renal failure and sepsis. The Biochemical journal.

[29] Libert DM, Nowacki AS, Natowicz MR (2018). Metabolomic analysis of obesity, metabolic syndrome, and type 2 diabetes: amino acid and acylcarnitine levels change along a spectrum of metabolic wellness. PeerJ..

[30] Brennan L, Hewage C, Malthouse JP, McBean GJ, An NMR (2003). study of alterations in [1-13C]glucose metabolism in C6 glioma cells by gliotoxic amino acids. Neurochemistry international.

[31] Han Q, Cai T, Tagle DA, Robinson H, Li J (2008). Substrate specificity and structure of human aminoadipate aminotransferase/kynurenine aminotransferase II. Biosci Rep.

[32] O’Neill E, Chiara Goisis R, Haverty R, Harkin A (2019). L-alpha-aminoadipic acid restricts dopaminergic neurodegeneration and motor deficits in an inflammatory model of Parkinson’s disease in male rats. Journal of neuroscience research.

[33] Takada M, Li ZK, Hattori T (1990). Astroglial ablation prevents MPTP-induced nigrostriatal neuronal death. Brain research.

[34] Haugstad TS, Langmoen IA (1997). L-alpha-aminoadipate reduces glutamate release from brain tissue exposed to combined oxygen and glucose deprivation. Journal of cerebral blood flow and metabolism: official journal of the International Society of Cerebral Blood Flow and Metabolism.

[35] Leandro, J. *et al*. DHTKD1 and OGDH display *in vivo* substrate overlap and form a hybrid ketoacid dehydrogenase complex (2019). 10.1093/hmg/ddaa037 PMC7206849 32160276

[36] Bunik VI, Fernie AR (2009). Metabolic control exerted by the 2-oxoglutarate dehydrogenase reaction: a cross-kingdom comparison of the crossroad between energy production and nitrogen assimilation. The Biochemical journal.

[37] Bunik VI, Tylicki A, Lukashev NV (2013). Thiamin diphosphate-dependent enzymes: from enzymology to metabolic regulation, drug design and disease models. The FEBS journal.

[38] Nemeria NS, Gerfen G, Yang L, Zhang X, Jordan F (2018). Evidence for functional and regulatory cross-talk between the tricarboxylic acid cycle 2-oxoglutarate dehydrogenase complex and 2-oxoadipate dehydrogenase on the l-lysine, l-hydroxylysine and l-tryptophan degradation pathways from studies *in vitro*. Biochim Biophys Acta Bioenerg.

[39] Winkel, B. S. J. In *Plant-derived Natural Products: Synthesis*, *Function*, *and Application* (eds A. E. Osbourn & V. Lanzotti) 195–208 (Springer-Verlag, New York, 2009).

[40] Zhou J (2018). A multipronged approach unravels unprecedented protein-protein interactions in the human 2-oxoglutarate dehydrogenase multienzyme complex. The Journal of biological chemistry.

[41] Williamson, D. H. & Brosnan, J. T. In *Methods of enymatic analysis* Vol. 4 (ed H.-U. Bergmeyer) 2266–2302 (Academic Press, Inc., 1974).

[42] Bunik VI (2005). Phosphonate analogues of alpha-ketoglutarate inhibit the activity of the alpha-ketoglutarate dehydrogenase complex isolated from brain and in cultured cells. Biochemistry.

[43] Araujo WL, Nunes-Nesi A, Trenkamp S, Bunik VI, Fernie AR (2008). Inhibition of 2-oxoglutarate dehydrogenase in potato tuber suggests the enzyme is limiting for respiration and confirms its importance in nitrogen assimilation. Plant physiology.

[44] Chenotherapy: questions - answers. *Med Chir Dig* **7**, 347–351 (1978). 672283

[45] Safronov, V. A. Diagnosis and treatment of toxic pulmonary edema at medical evacuation stages. *Voen Med Zh*, 29–34 (1978). 676157

[46] Sirina LK, Glotova TP, Kutsemilova AP (1978). Functional state of the sympathetic-adrenal system in meningococcal infections. Zh Nevropatol Psikhiatr Im S S Korsakova.

[47] Wiczer BM, Bernlohr DA (2009). A novel role for fatty acid transport protein 1 in the regulation of tricarboxylic acid cycle and mitochondrial function in 3T3-L1 adipocytes. Journal of lipid research.

[48] Bennett MJ (1990). The laboratory diagnosis of inborn errors of mitochondrial fatty acid oxidation. Ann Clin Biochem.

[49] Lord, R. S. & Bralley, J. A. *Nitrogen Metabolism in Plants*. 2 edn, (Metamerix Institute, 2008).

[50] Marlaire S, Van Schaftingen E, Veiga-da-Cunha M (2014). C7orf10 encodes succinate-hydroxymethylglutarate CoA-transferase, the enzyme that converts glutarate to glutaryl-CoA. J Inherit Metab Dis.

[51] Xu J (2018). Genetic identification of leptin neural circuits in energy and glucose homeostases. Nature.

[52] Tsuneki H, Sasaoka T, Sakurai T (2016). Sleep Control, GPCRs, and Glucose Metabolism. Trends Endocrinol Metab.

[53] Kootte RS (2017). Improvement of Insulin Sensitivity after Lean Donor Feces in Metabolic Syndrome Is Driven by Baseline Intestinal Microbiota Composition. Cell metabolism.

[54] Halson SL (2014). Sleep in elite athletes and nutritional interventions to enhance sleep. Sports Med.

[55] Feng AL (2017). Paracrine GABA and insulin regulate pancreatic alpha cell proliferation in a mouse model of type 1 diabetes. Diabetologia.

[56] Plubell DL (2018). GM-CSF driven myeloid cells in adipose tissue link weight gain and insulin resistance via formation of 2-aminoadipate. Scientific reports.

[57] Santos SS (2006). Inhibitors of the alpha-ketoglutarate dehydrogenase complex alter [1-13C]glucose and [U-13C]glutamate metabolism in cerebellar granule neurons. Journal of neuroscience research.

[58] Drexel H (2007). Nicotinic acid in the treatment of hyperlipidaemia. Fundam Clin Pharmacol.

[59] Goldberg RB (2016). Effects of Extended-Release Niacin Added to Simvastatin/Ezetimibe on Glucose and Insulin Values in AIM-HIGH. Am J Med.

[60] Liu D, Wang X, Kong L, Chen Z (2015). Nicotinic acid regulates glucose and lipid metabolism through lipid independent pathways. Curr Pharm Biotechnol.

[61] Pires JAA (2016). Effects of abomasal infusion of nicotinic acid on responses to glucose and beta-agonist challenges in underfed lactating cows. J Dairy Sci.

[62] Goldberg RB, Jacobson TA (2008). Effects of niacin on glucose control in patients with dyslipidemia. Mayo Clin Proc.

[63] Ding Y, Li Y, Wen A (2015). Effect of niacin on lipids and glucose in patients with type 2 diabetes: A meta-analysis of randomized, controlled clinical trials. Clin Nutr.

[64] Wahlberg G, Walldius G, Efendic S (1992). Effects of nicotinic acid on glucose tolerance and glucose incorporation into adipose tissue in hypertriglyceridaemia. Scand J Clin Lab Invest.

[65] Xu WY (2019). 2-Aminoadipic acid protects against obesity and diabetes. The Journal of endocrinology.

[66] Lim J (2014). Dual mode action of mangiferin in mouse liver under high fat diet. PloS one.

[67] Timmons JA (2018). A coding and non-coding transcriptomic perspective on the genomics of human metabolic disease. Nucleic acids research.

[68] Tsepkova PM (2017). Thiamine Induces Long-Term Changes in Amino Acid Profiles and Activities of 2-Oxoglutarate and 2-Oxoadipate Dehydrogenases in Rat Brain. *Biochemistry*. Biokhimiia.

[69] Wagner Tristan, Boyko Alexandra, Alzari Pedro M., Bunik Victoria I., Bellinzoni Marco (2019). Conformational transitions in the active site of mycobacterial 2-oxoglutarate dehydrogenase upon binding phosphonate analogues of 2-oxoglutarate: From a Michaelis-like complex to ThDP adducts. Journal of Structural Biology.

[70] Rosi A (2015). 1) H NMR spectroscopy of glioblastoma stem-like cells identifies alpha-aminoadipate as a marker of tumor aggressiveness. NMR in biomedicine.

[71] Hallen A, Jamie JF, Cooper AJ (2013). Lysine metabolism in mammalian brain: an update on the importance of recent discoveries. Amino acids.

[72] Pena IA (2016). Simultaneous detection of lysine metabolites by a single LC-MS/MS method: monitoring lysine degradation in mouse plasma. SpringerPlus.

[73] Pena IA (2017). Mouse lysine catabolism to aminoadipate occurs primarily through the saccharopine pathway; implications for pyridoxine dependent epilepsy (PDE). *Biochimica et biophysica acta*. Molecular basis of disease.

[74] Posset R (2015). Understanding cerebral L-lysine metabolism: the role of L-pipecolate metabolism in Gcdh-deficient mice as a model for glutaric aciduria type I. Journal of inherited metabolic disease.

[75] Papatheodorou I (2018). Expression Atlas: gene and protein expression across multiple studies and organisms. Nucleic acids research.

[76] Petryszak R (2016). Expression Atlas update–an integrated database of gene and protein expression in humans, animals and plants. Nucleic acids research.

[77] Sauer SW (2011). Therapeutic modulation of cerebral L-lysine metabolism in a mouse model for glutaric aciduria type I. Brain: a journal of neurology.

[78] Shibata K (2018). Organ Co-Relationship in Tryptophan Metabolism and Factors That Govern the Biosynthesis of Nicotinamide from Tryptophan. Journal of nutritional science and vitaminology.

[79] Kulikova VA, Gromyko DV, Nikiforov AA (2018). The Regulatory Role of NAD in Human and Animal Cells. *Biochemistry*. Biokhimiia.

[80] Nikiforov A, Kulikova V, Ziegler M (2015). The human NAD metabolome: Functions, metabolism and compartmentalization. Critical reviews in biochemistry and molecular biology.

[81] Shibata Katsumi, Yamazaki Marika, Matsuyama Yukiyo (2016). Urinary excretion ratio of xanthurenic acid/kynurenic acid as a functional biomarker of niacin nutritional status. Bioscience, Biotechnology, and Biochemistry.

[82] Kim BJ (2008). Generation of nicotinic acid adenine dinucleotide phosphate and cyclic ADP-ribose by glucagon-like peptide-1 evokes Ca2+ signal that is essential for insulin secretion in mouse pancreatic islets. Diabetes.

[83] Lee HC (2012). Cyclic ADP-ribose and nicotinic acid adenine dinucleotide phosphate (NAADP) as messengers for calcium mobilization. The Journal of biological chemistry.

[84] Park KH (2013). Autocrine/paracrine function of nicotinic acid adenine dinucleotide phosphate (NAADP) for glucose homeostasis in pancreatic beta-cells and adipocytes. The Journal of biological chemistry.

[85] Chen T (2016). Tryptophan Predicts the Risk for Future Type 2 Diabetes. PloS one.

[86] Stanley CJ, Perham RN (1980). Purification of 2-oxo acid dehydrogenase multienzyme complexes from ox heart by a new method. The Biochemical journal.

[87] Bradford MM (1976). A rapid and sensitive method for the quantitation of microgram quantities of protein utilizing the principle of protein-dye binding. Analytical biochemistry.

[88] Zor T, Selinger Z (1996). Linearization of the Bradford protein assay increases its sensitivity: theoretical and experimental studies. Analytical biochemistry.

[89] Johnson, D. & Lardy, H. A. In *Estabrook*, *R*., *Pullmam*, *M*. *N*.*Y*. Vol. 10 94–101 (London: Academic Press, 1967).

[90] Mkrtchyan G (2015). Molecular mechanisms of the non-coenzyme action of thiamin in brain: biochemical, structural and pathway analysis. Scientific reports.

[91] Bunik VI (2015). Specific inhibition by synthetic analogs of pyruvate reveals that the pyruvate dehydrogenase reaction is essential for metabolism and viability of glioblastoma cells. Oncotarget.

[92] Bunik V (2016). Inhibition of mitochondrial 2-oxoglutarate dehydrogenase impairs viability of cancer cells in a cell-specific metabolism-dependent manner. Oncotarget.

[93] Lisec J, Schauer N, Kopka J, Willmitzer L, Fernie AR (2006). Gas chromatography mass spectrometry-based metabolite profiling in plants. Nature protocols.

[94] Edgar R, Domrachev M, Lash AE (2002). Gene Expression Omnibus: NCBI gene expression and hybridization array data repository. Nucleic acids research.

[95] Barrett T (2013). NCBI GEO: archive for functional genomics data sets–update. Nucleic acids research.

[96] Mkrtchyan G, Graf A, Bettendorff L, Bunik V (2016). Cellular thiamine status is coupled to function of mitochondrial 2-oxoglutarate dehydrogenase. Neurochemistry international.

[97] Wang M (2012). PaxDb, a database of protein abundance averages across all three domains of life. Molecular & cellular proteomics: MCP.

[98] Wang M, Herrmann CJ, Simonovic M, Szklarczyk D, von Mering C (2015). Version 4.0 of PaxDb: Protein abundance data, integrated across model organisms, tissues, and cell-lines. Proteomics.

[99] Kolker E (2012). MOPED: Model Organism Protein Expression Database. Nucleic acids research.

[100] Montague E (2014). MOPED 2.5–an integrated multi-omics resource: multi-omics profiling expression database now includes transcriptomics data. Omics: a journal of integrative biology.

[101] Geiger T (2013). Initial quantitative proteomic map of 28 mouse tissues using the SILAC mouse. Molecular & cellular proteomics: MCP.

[102] Deshmukh AS (2015). Deep proteomics of mouse skeletal muscle enables quantitation of protein isoforms, metabolic pathways, and transcription factors. Molecular & cellular proteomics: MCP.

[103] Kislinger T (2006). Global survey of organ and organelle protein expression in mouse: combined proteomic and transcriptomic profiling. Cell.

[104] Huttlin EL (2010). A tissue-specific atlas of mouse protein phosphorylation and expression. Cell.

[105] Kruger M (2008). SILAC mouse for quantitative proteomics uncovers kindlin-3 as an essential factor for red blood cell function. Cell.

[106] Shi R (2007). Analysis of the mouse liver proteome using advanced mass spectrometry. Journal of proteome research.

[107] Meierhofer D, Halbach M, Sen NE, Gispert S, Auburger G (2016). Ataxin-2 (Atxn2)-Knock-Out Mice Show Branched Chain Amino Acids and Fatty Acids Pathway Alterations. Molecular & cellular proteomics: MCP.

[108] Hasan M (2019). Quantitative Proteome Analysis of Brain Subregions and Spinal Cord from Experimental Autoimmune Encephalomyelitis Mice by TMT-Based Mass Spectrometry. Proteomics.

[109] Wang H (2006). Characterization of the mouse brain proteome using global proteomic analysis complemented with cysteinyl-peptide enrichment. Journal of proteome research.

[110] Trofimova LK (2012). Consequences of the alpha-ketoglutarate dehydrogenase inhibition for neuronal metabolism and survival: implications for neurodegenerative diseases. Current medicinal chemistry.

[111] Luedemann A, Strassburg K, Erban A, Kopka J (2008). TagFinder for the quantitative analysis of gas chromatography–mass spectrometry (GC-MS)-based metabolite profiling experiments. Bioinformatics.

[112] Kopka J (2005). GMD@CSB.DB: the Golm Metabolome Database. Bioinformatics.

[113] Schauer N (2005). GC-MS libraries for the rapid identification of metabolites in complex biological samples. FEBS letters.

[114] Bunik V (1999). Interaction of thioredoxins with target proteins: role of particular structural elements and electrostatic properties of thioredoxins in their interplay with 2-oxoacid dehydrogenase complexes. *Protein science: a publication of the Protein*. Society.

[115] Canto C, Menzies KJ, Auwerx J (2015). NAD(+) Metabolism and the Control of Energy Homeostasis: A Balancing Act between Mitochondria and the Nucleus. Cell metabolism.

[116] Katsyuba E, Auwerx J (2017). Modulating NAD(+) metabolism, from bench to bedside. The EMBO journal.

