# Supplementary material for: Synthetic analogues of 2-oxo acids discriminate metabolic contribution of the 2-oxoglutarate and 2-oxoadipate dehydrogenases in mammalian cells and tissues
Source: Sci Rep. 2020 Feb 5;10:1886. doi: 10.1038/s41598-020-58701-4 (PMC7002488; doi:10.1038/s41598-020-58701-4)
Supplement: Supplementary file 1 — Supplementary information. [file 41598_2020_58701_MOESM1_ESM.docx]

*Supporting Information for*

**Synthetic analogues of 2-oxo acids discriminate metabolic contribution of the 2-oxoglutarate and 2-oxoadipate dehydrogenases in mammalian cells and tissues.**

Artem V. Artiukhov^1, 2^, Aneta Grabarska^3^, Ewelina Gumbarewicz^3^, Vasily A. Aleshin^1, 2^, Thilo Kähne^4^, Toshihiro Obata^5, †^, Alexey V. Kazantsev^6^, Nikolay V. Lukashev^6^, Andrzej Stepulak^3^, Alisdair R. Fernie^5^, Victoria I. Bunik^1, 2,^ *


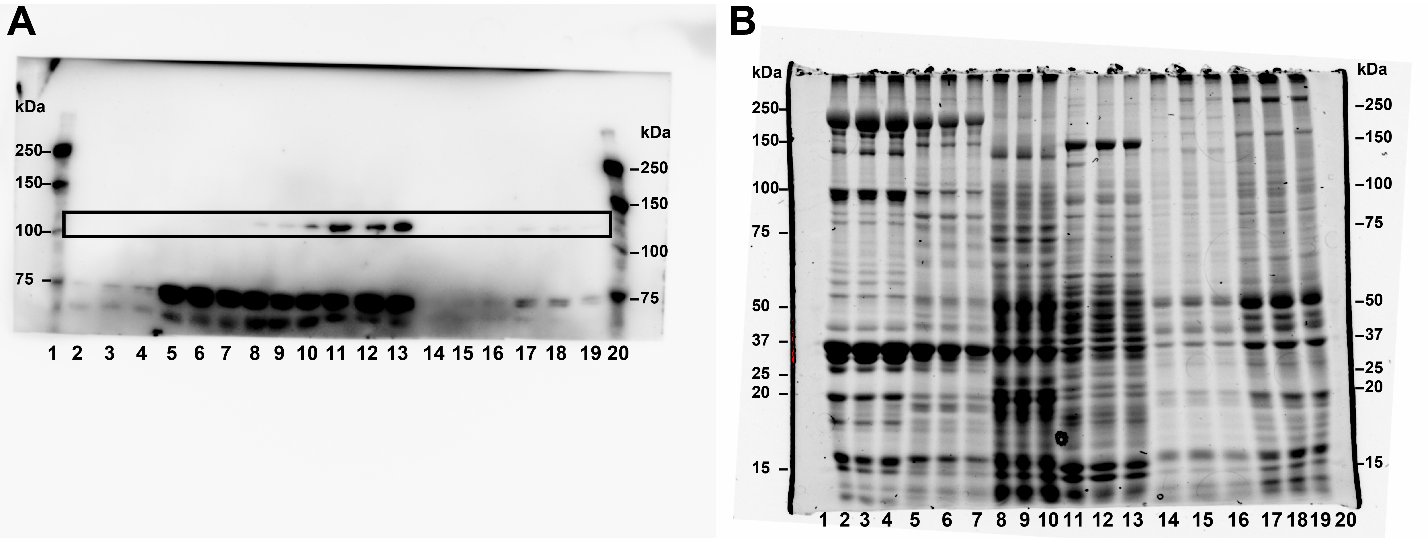


**Supplementary Figure 1. Raw results of western-blot analysis of DHTKD1 expression in rat tissues.** (A) An image obtained after staining of cropped PVDF-membrane, where the tissue samples were transferred after SDS-PAGE, with anti-DHTKD1 primary antibodies and HRP-linked secondary antibodies (described in Materials and Methods) followed by chemiluminescence visualization. The membrane part, corresponding to the molecular mass of native DHTKD1 (100-110 kDa) and antibody manufacturer’s recommendations, was used for calculation of DHTKD1 abundance presented in Fig. 1A. The part is marked by black line. (B) An image obtained after the SDS-PAGE of the same tissue samples followed by the protein visualization in the stain-free mode (see Materials and Methods). All tissue samples are in triplicates. Lanes numbered 1 and 20 correspond to protein standards, 2-4 – to skeletal muscle, 5-7 – to heart, 8-10 – to kidney, 11-13 – to liver, 14-16 – to spinal cord, 17-19 – to brain. The total protein staining of the gel indicates that the protein content in spinal cord is low, compared to other samples, despite its expected similarity, based on our and others (Banay-Schwartz et al., 1992) experience.


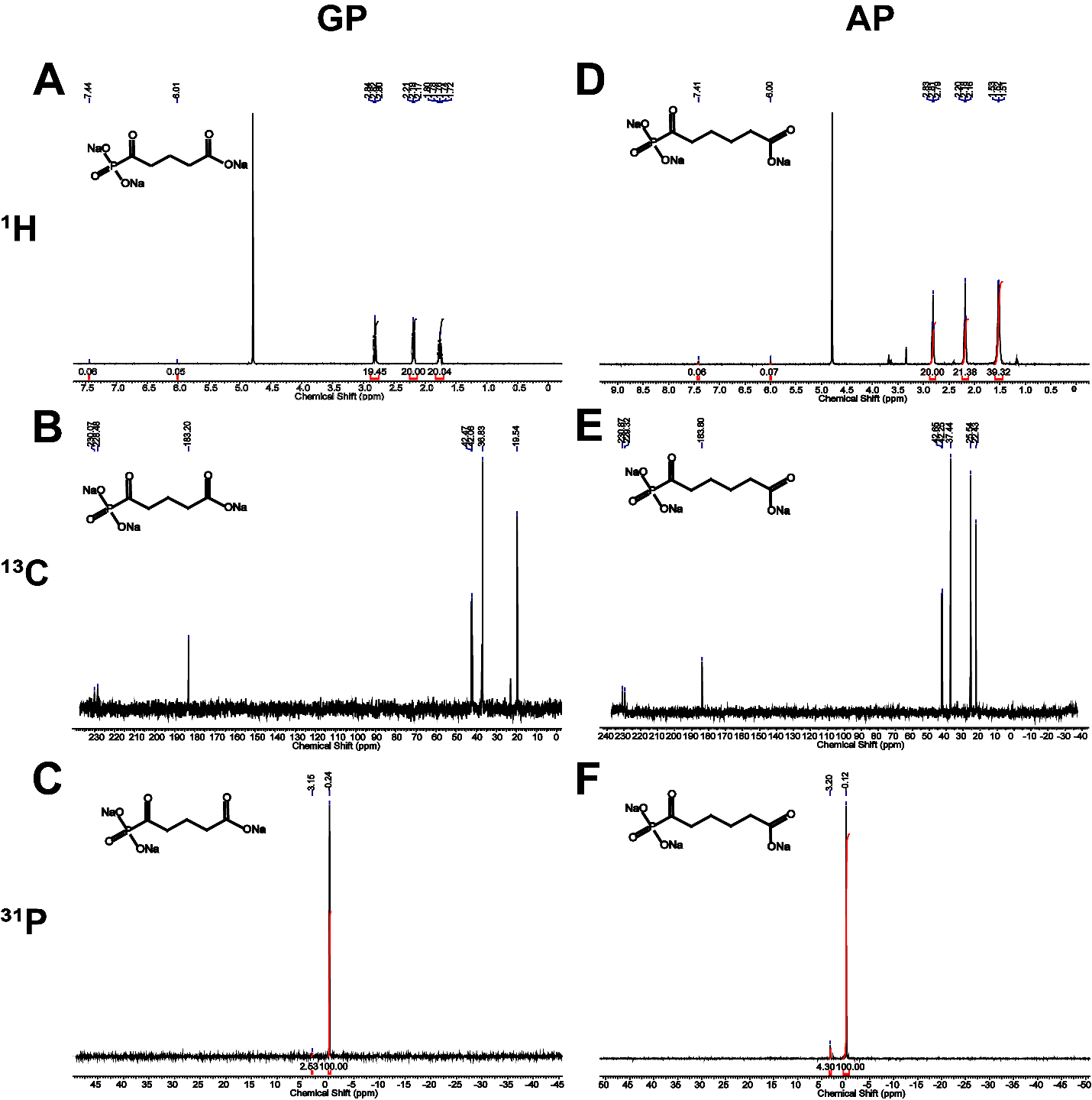


**Supplementary Figure 2. NMR spectra of synthesized GP (A-C) and AP (D-F) preparations.** ^1^H (A, D), ^13^C (B, E) and ^31^P (C, F) spectra were recorded at 400, 100.6 and 161.9 MHz, respectively. D_2_O was used as a standard.


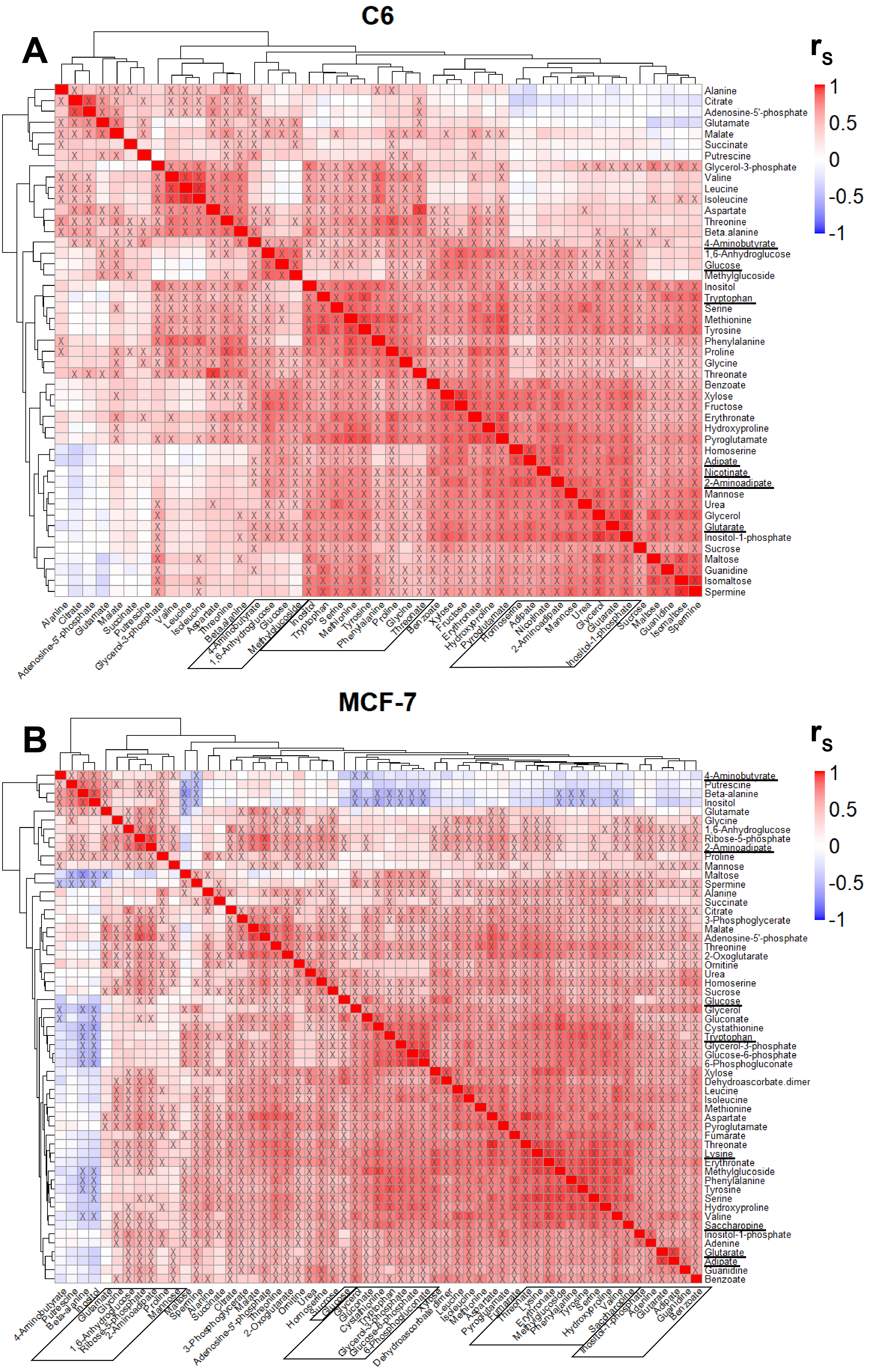


**Supplementary Figure 3. Correlation between the metabolite levels in C6 (A) and MCF-7 (B) cells.** Spearman’s correlation coefficients (r_S_) for the levels of metabolites from all the samples (Ctrl and SP-, GP- and AP-treated) in one cell line, together with the p-values of the correlations were calculated in GraphPad Prism v 8.0 and visualized using *pheatmap* package in R (<https://cran.r-project.org/web/packages/pheatmap/>). Clustering of experimental groups and metabolites uses Manhattan as a distance measure and WPGMA as an agglomeration method. X indicates significant correlations (p ≤ 0.05).

**References**

Banay-Schwartz, M., Kenessey, A., DeGuzman, T., Lajtha, A., Palkovits M. Protein content of various regions of rat brain and adult and aging human brain. AGE 15, 51–54 (1992) https://doi.org/10.1007/BF02435024
